# Cyclopropane xenolipids resemble monounsaturated fatty acids and modulate peroxisome proliferator-activated receptors

**DOI:** 10.1016/j.jlr.2025.100896

**Published:** 2025-09-05

**Authors:** Jean Debédat, Lorena Pastor, Trina A. Knotts, Jordan G. Pitman, Kristine Griffett, Sean H. Adams

**Affiliations:** 1 Department of Surgery, University of California Davis School of Medicine, Sacramento, CA, USA; 2 Center for Alimentary and Metabolic Science, University of California Davis School of Medicine, Sacramento, CA, USA; 3 Department of Anatomy, Physiology, and Pharmacology, Auburn University College of Veterinary Medicine, Auburn, AL, USA

**Keywords:** xenometabolites, PPAR, in silico, cyclopropane FAs, xenolipids, MUFAs, saturated FAs

## Abstract

Cyclopropane FAs (CpFAs) are members of the mammalian lipidome, originating from the diet and gut microbial metabolism. Despite being fully saturated, conformational modeling of CpFAs from C12 to C24 in length revealed that they are bent lipids sharing structural similarities with MUFAs. We therefore hypothesized that CpFAs might share some bioactivities with MUFAs. We modeled and docked a total of 429 known and theoretical CpFAs, MUFAs, and saturated lipids into PPAR α, δ, and γ nuclear receptor structures. CpFAs showed unique spatial binding patterns, especially with PPARδ. In vitro, several CpFAs bound PPARα and δ with potencies comparable to dietary MUFAs, whereas in 3T3-L1 preadipocytes, they upregulated the pan-PPAR target gene *Angptl4*, indicating downstream functional engagement. These findings suggest that CpFAs share some structural and functional aspects with MUFAs and represent an under-recognized class of metabolically relevant food- and gut-derived lipids.

The gastrointestinal (GI) tract is at the nexus of two major factors that regulate human physiology: diet and the gut microbiota. The GI lumen harbors thousands of molecules derived from microbes, microbial metabolism, foods, and the host. A substantial portion of microbe-derived “nonself” metabolites (xenometabolites) can enter the circulation with the potential to impact host physiology in a variety of tissues. Based on correlations between specific human fecal bacteria to blood metabolites (1, 2) and large differences in blood metabolites between germ-free and specific pathogen-free normal mice (3, 4), we estimate that no less than 20% of blood metabolites derive from GI tract bacterial metabolism. Most xenometabolites or suspected xenometabolites remain nonannotated, “unknown” compounds (e.g., see Ref. (5)). While a great deal of research has historically focused on small molecules produced from the breakdown of dietary fibers, such as short-chain FAs, a variety of other xenometabolites with reported health-modulating properties have been described: for example, xenolipids such as hydroxy lipids (6), conjugated linoleic acid (7), branched-chain FAs (8), and amino acid derivatives, such as imidazole propionate (9) and indoles (10, 11).

Among the underexplored landscape of xenolipids are the cyclopropane FAs (CpFAs). First identified 75 years ago by Hoffmann *et al.* (12) in bacterial cultures, CpFAs are saturated lipids featuring a cyclopropane ring added across the double bond of MUFAs by the action of cyclopropane FA synthase (13). Although initially discovered in *Lactobacillus arabinosus* (12) many bacteria, algae, fungi, and plants possess the enzymatic machinery to produce CpFAs (14, 15, 16). In bacteria, CpFAs serve functional roles in stress adaptation, for example, by enhancing membrane resistance to antibiotics, acids, or other environmental stresses, and facilitate gut colonization in rodents (17, 18). Some even-chain CpFAs, such as lactobacillic acid (11,12-P-19:0; also known as *cis*-11,12-methylene-octadecanoic acid), dihydrosterculic acid (9,10-P-19:0; also known as *cis*-9,10-methylene-octadecanoic acid), and “CPOA2H” (9,10-P-17:0; also known as *cis*-9,10-methylene-hexadecanoic acid), have been identified in animal-based foods (meat, fish, and dairy) (19, 20) and in plant-based food components, such as cottonseeds, certain mushrooms, and algae (16, 21, 22). An Italian study estimated that daily intake of two specific CpFA species, lactobacillic and dihydrosterculic acids, was around 12 mg/day (22). Given the growing number of identified CpFAs and the likelihood that many more remain uncharacterized, actual dietary exposure is likely to be considerably higher. The contribution of the gut microbiota to host CpFA status is less clear, but in one study, Colossimo *et al.* (23) showed that the introduction of a bacterial consortia with cyclopropane FA-positive bacteria in germ-free mice led to a rise in the cecal abundance of 9,10-P-17:0 that was absent prior to bacterial inoculation. We recently characterized the novel odd-chain CpFAs 11,12-P-16:0 (also known as *cis*-11,12-methylene-pentadecanoic acid) and 13,14-P-18:0 (also known as *cis*-13,14-methylene-heptadecanoic acid) in porcine stool and blood biospecimens, and tissue flux analyses in growing pigs support a GI source (24).

Regardless of CpFA origins (dietary, in situ microbial metabolism, or both), an important point is that CpFAs are natural components of the mammalian lipidome in blood and at least some tissues, such as adipose, heart, and liver (20, 24, 25, 26, 27), suggesting that members of this lipid class can impact physiology. Indeed, emerging research indicates potential associations between metabolic health status and the biology of some CpFAs. For instance, the serum level of 9,10-P-17:0 was found to be 40% higher in adults with obesity compared with persons without obesity and normalized after weight loss (20). The byproduct of incomplete beta-oxidation of an odd-chain long-chain CpFA (likely C15Δ1 *cis*-11 and/or C17Δ1 *cis*-13), *cis*-3,4-methylene-heptanoylcarnitine, is abundant in human blood relative to other medium-chain acylcarnitines, and concentrations dropped significantly with a weight loss and exercise intervention that improved insulin sensitivity and cardiometabolic fitness (28). A recent study that combined in silico modeling and biochemical binding assays demonstrated that lactobacillic acid (C18Δ1 *cis*-11) and a cyclopropene FA (sterculic acid) can act as PPARγ ligands (29). However, potential interactions of other CpFAs with PPARγ were not evaluated, and it is not known if CpFAs can interact with other PPAR isoforms (PPARα and PPARδ).

Another aspect of CpFAs that has not been considered is their potential structural similarity to MUFAs, despite CpFAs technically being a type of saturated FA (SFA). We hypothesize that the cyclopropane ring confers a “bend” in the molecule, analogous to the double bond of MUFAs and distinct from the more linear structure of SFAs. If true, we reason that CpFAs and MUFAs of identical chain length should share some structural properties and functional outcomes with respect to PPAR binding. Herein, we addressed these knowledge gaps by testing binding characteristics of five different CpFAs—including two novel odd-chain CpFAs—to human PPARα, PPARδ, and PPARγ (NR1C1, NR1C2, and NR1C3, respectively). In addition, functional experiments in murine 3T3-L1 cells determined if the xenolipids activate PPAR-associated target gene expression. Finally, we characterized the impacts of chain length, unsaturation, and methylene group location on PPAR binding and physicochemical properties across hundreds of known and theoretical FAs.

## Materials and Methods

### Docking studies in silico

All in silico experiments were performed using Maestro, version 14.2.118, from the Schrödinger software suite (version 2024-4).

#### Human PPAR structures

A total of 12 crystal structures of the ligand-binding domains (LBDs) of PPARα, PPARδ, and PPARγ, all with a resolution of 2 Å or better, were obtained from the RCSB Protein Data Bank (https://www.rcsb.org/) and imported into Maestro. Unbound structures of each PPAR isoform were generated by Google DeepMind and downloaded from the AlphaFold Protein Structure Database (https://alphafold.ebi.ac.uk/). Details about the characteristics of these 15 structures are available in supplemental Table S1. For structures containing multiple chains, the B chain and all associated atoms were removed before protein preparation.

#### Protein preparations

The Protein Preparation Wizard (Maestro) was used to prepare and optimize the structures and to fill in missing side chains, with the standard preprocessing protocol. Briefly, this included assigning bond orders, adding hydrogens, creating missing disulfide bonds, and removing water molecules located more than 5 Å from the cocrystallized ligand. The optimization of the crystal structure was carried out by applying the OPLS4 (optimized potential for liquid simulation 4) force field (30), imposing a 0.3 Å root mean square deviation (RMSD) limit as the constraint. The PROPKA program of the Protein Preparation Wizard was used to generate the protonation and ionization state of amino acids at physiological pH (7.4 ± 2.0).

#### Protein alignments

Prepared protein structures were aligned in pairs using the Protein Structure Alignment (Maestro), with reference residues set as “(protein) and not ligand,” aligning only residues also found in the reference. Each structure was iteratively used as the reference until alignment scores and RMSD values were gathered for all pairs.

#### Receptor grids

Grids were prepared for each receptor structure to perform virtual docking within a constrained area around the binding domain. A 20 Å box was centered around the cocrystallized ligand within each receptor. To position grid boxes on the AlphaFold structures (which lack bound ligands), all structures were aligned, and the average coordinates of the grid boxes of the four crystal structures were used for the AlphaFold structures, for each PPAR isoform independently.

#### Large-scale lipid ligand library preparation

To examine and compare the estimated binding potential of SFAs, MUFAs, and CpFAs toward PPARs, all possible lipids from these classes with carbon backbones ranging from 12 to 24 atoms were evaluated. For MUFAs and CpFAs, all possible positions for a single double bond and cyclopropane ring were considered in the *cis* conformation. This in silico work included a total of 429 known and theoretical lipids (13 SFAs, 208 MUFAs, and 208 CpFAs). Detailed structures and SMILES representation of the 429 lipids can be found in supplemental Table S2. The 2D structures of all lipid ligands were imported into Maestro using isomer-explicit SMILES representations and subsequently prepared using the LigPrep tool. Molecular states were generated at neutral pH (7.0 ± 2.0) using Epik Classic, with desalted outputs, tautomers allowed, and chiralities preserved (default settings). The OPLS4 force field was used throughout the LigPrep process.

#### Lipid structural and conformational analysis

Data exploring the structural diversity and compactness of all 429 lipids were generated using Schrödinger's ConfGen tool. A maximum of 999 low-energy, nonredundant conformers were produced per lipid, yielding a total of 428,571 conformers. Of note, the system failed to generate up to 999 nonredundant conformers for some of the smaller FAs with 12–14 carbon backbones (supplemental Fig. S1). Atomic coordinates (x, y, and z) for all carbon atoms were extracted using *StructureReader* from the *schrodinger.structure* Python module. End-to-end distances were measured between both terminal carbons in each conformer to assess overall molecular extension. The end-to-end Euclidean distance (d_ee_) formula is d_ee_ = sqrt((x_last_ - x_x1_)^2^ + (y_last_ - y_x1_)^2^ + (z_last_ - z_x1_)^2^), where (x_x1_, y_x1_, z_x1_) are the atomic coordinates of the first carbon atom (base of the carboxyl group) and (x_last_, y_last_, z_last_) are the coordinates of the last carbon atom (end of the lipid tail) along the molecular backbone. The radius of gyration was determined to quantify conformer compactness (with higher values indicating more extended structures and lower values indicating more compacted structures), calculated as: R_g_ = sqrt((1/N) ∗Σ [(xᵢ - x¯)^2^ + (yᵢ - ȳ)^2^ + (zᵢ - z¯)^2^]), where N is the number of carbon atoms in the conformer, (xᵢ, yᵢ, zᵢ) are the atomic coordinates of individual carbons, and (x¯, ȳ, z¯) represent the center of mass of the conformer. Principal component analysis (PCA) was applied to the atomic coordinates to identify trends in structural diversity, and the convex hull area (using convhulln() from the geometry R package) was computed per conformer to quantify conformational diversity, with larger areas indicating greater flexibility and spatial spread.

#### Virtual screening

Virtual docking was performed using the Virtual Screening Workflow (VSW) tool in Maestro. Virtual Screening Workflow was performed, including all prepared ligands and on three receptor grids per run. The docking process applied extra precision settings with Epic state penalties. The van der Waals (vdW) radii for nonpolar atoms were scaled by a factor of 0.8, with a partial charge cutoff set at 0.15. Ligand flexibility was allowed during docking, although nonplanar conformations of amide bonds were penalized. Postdocking minimization was then conducted, and up to five poses with the best docking scores (estimates of predicted ligand-protein binding affinity) were retained per compound state. Finally, binding energy (BE) predictions were refined using molecular mechanics-generalized born surface area (MM-GBSA), which relies on Prime's solvation model (30). This approach calculates the free energy of binding by integrating molecular mechanics, the generalized Born model, and solvent accessibility, with results expressed in kilocalories per mole. Since MM-GBSA BEs approximate the free energy of binding, a more negative value corresponds to stronger predicted binding affinity.

#### FA positions within PPAR LBDs

A total of 100 low-energy, nonredundant conformers were produced for each lipid included in structural modeling work (supplemental Table S3), yielding a total of 1,400 conformers. Each conformer was docked within the LBD area of the AlphaFold PPAR structures. The docking process applied extra precision settings with Epic state penalties. The vdW radii for nonpolar atoms were scaled by a factor of 0.8, with a partial charge cutoff set at 0.15. Ligand flexibility was allowed during docking, although nonplanar conformations of amide bonds were penalized. Postdocking minimization was then conducted, and up to 25 poses with the best docking scores were retained per compound state. After docking, only poses for which the minimization successfully converged were retained for further analysis, ensuring that all selected poses represented energetically stable conformations. A total of 10,285, 10,816, and 10,054 poses were retained for human PPARα, PPARδ, and PPARγ, respectively. As the number of poses was not equivalent across lipids, these poses were subsampled (with seed) to 461 poses per lipid for PPARα (total: 6,454 poses), 371 for PPARδ (total: 5,194 poses), and 407 for PPARγ (total: 5,698 poses). The Cartesian coordinates of the carboxyl group oxygen atoms of all 17,346 lipid poses were extracted using *StructureReader* from the *schrodinger.structure* Python module. For each lipid, the coordinates of both oxygens were averaged to simplify visualizations.

#### Clustering of lipid positions

K-means clusters were generated for each projection, whether per PPAR isoform (see Results section) or per PPAR isoform and lipid category (supplemental Fig. S11). The appropriate number of clusters was determined using a combination of the elbow method and silhouette analysis. For the elbow method, we calculated the total within-cluster sum of squares (WSS) across a range of cluster numbers (1 ≤ k ≤ 10) using the kmeans() function from the stats package in R. The optimal k was initially estimated by identifying an inflection point in the WSS curve, where the second derivative dropped below a threshold of 10,000. We then used silhouette analysis (via silhouette() from the cluster R package) to evaluate clustering quality, favoring solutions with higher average silhouette widths (indicating greater cohesion within clusters and clearer separation between clusters). When the elbow and silhouette results differed, the final k was chosen based on both cluster separation and interpretability. To assess the impact of clusters and lipid categories on docking potential, we compared Glide Emodel docking scores (expressed in kilocalories per·mole). Glide Emodel was used as it better reflects pose quality and initial docking energetics, making it more appropriate for evaluating differences across a large number of lipid poses.

#### Residue interaction fingerprinting

Residue-level interaction fingerprints were generated using Schrödinger’s interactionfp module. Interactions were computed within a 4.0 Å cutoff from each ligand pose, aggregating all interaction types, including contact, backbone, side chain, polar, hydrophobic, acceptor, donor, aromatic, and charged interactions. For each lipid-residue pair, the total number of interactions was normalized by the total number of conformers evaluated for that lipid, providing a normalized measure of interaction frequency.

### In vitro binding studies

LanthaScreen® time-resolved fluorescence resonance energy transfer (TR-FRET) coactivator assay kits for human PPARα (ref. no.: PV4684; ThermoFisher Scientific, Waltham, MA), PPARδ (ref. no.: PV4685; ThermoFisher Scientific), and PPARγ (ref. no.: PV4548; ThermoFisher Scientific) were performed according to the manufacturer’s instructions using low-binding black 384-well plates (ref. no.: 4514; Corning, New York, NY) and a SpectraMax i3x plate reader (Molecular Devices, San Jose, CA) equipped with a TR-FRET B/G custom cartridge (filters for excitation at 340 nm and emissions at 495 nm and 520 nm). Each assay included a fluorescein-labeled coactivator peptide specific to the target receptor: PGC1 α for PPARα, C33 for PPARδ, and TRAP220/DRIP-2 for PPARγ.

Stock solutions (100×) of the tested FAs (supplemental Table S3) and known PPAR agonists (supplemental Table S4) were prepared in DMSO (≥99.7% purity, ref. no.: BP231; Fisher Scientific, Waltham, MA) and subsequently diluted by half across 12 series. Briefly, the appropriate volume of DMSO was injected directly through the vial’s septum using a syringe. For lipids bought in larger quantities (>100 mg), roughly ∼20 mg was weighed on a high-precision scale (ref. no.: ML54T/00; Mettler Toledo, Columbus, OH) before adding the proper volume of DMSO to reach 100× concentrations. In all scenarios, the vials were vigorously mixed to guarantee solubilization. Serial dilutions were performed in DMSO-resistant 96-well plates (ref. no.: 249944; ThermoFisher Scientific). Finally, stocks were diluted in the kit’s buffer, resulting in a final DMSO concentration of 5% (by volume) per well. As some test lipids were purchased already solubilized in ethanol, ethanol was added appropriately to all stocks so that all lipid preparations were in the same amount of ethanol and DMSO to harmonize conditions.

Appropriate controls (no agonist, no agonist plus no LBD) were included in all experiments to ensure reproducibility and normalization across replications. All results were normalized, with 0% binding representing the average 495/520 nm ratio of the no agonist control and 100% representing the average ratio of the agonist control at the highest concentration. The 495 nm signal corresponds to the terbium donor emission, and the 520 nm signal reflects energy transfer to the fluorescein-labeled coactivator peptide upon binding to the LBD. The resulting 520/495 ratio reflects ligand-induced receptor activation and is used to correct for well-to-well variation in donor intensity. Each experiment was performed in quadruplicate for accuracy and reproducibility and independently repeated two to five times. Nonlinear four-parameter logistic regressions were performed in R using the drm() function from the drc package. EC_50_ and 95% confidence intervals were computed using coef() and confint() from the stats package, respectively.

### Cell culture model for PPAR target gene expression

3T3-L1 murine preadipocytes (ref. no.: CL-173, batch no.: 70051703) were purchased from the ATCC (Manassas, VA) and used between passages 8 and 22. Cells were cultured in high-glucose DMEM containing pyruvate and glutamine (ref. no.: 11995065; Gibco, Waltham, MA) supplemented with 10% volume/volume FBS (ref. no.: 16000044, lot no.: 2405463RP; Gibco), 10,000 U of penicillin and streptomycin (ref. no.: 15140122; Gibco, Waltham, MA) and supplemented with 1% L-alanyl-L-glutamine dipeptide (ref. no.: 35050061; Gibco, Waltham, MA). The cells were maintained in a 5% CO_2_ incubator at 37°C.

#### Preparation of lipids and PPAR agonists and antagonists for cell culture

All FAs described in supplemental Table S3 were diluted in ethanol to make 100 mM stock solutions, aliquoted, and stored at −20°C. Nonlipid PPAR agonists and antagonists (supplemental Table S4) were diluted in DMSO to 10 mM stock solutions, aliquoted, and stored at −20°C.

#### Cell culture treatment paradigm

3T3-L1 cells were released from the dish using 0.25% trypsin-EDTA (ref. no.: 25200056; Gibco, Waltham, MA) and were counted using a Countess II (Life Technologies, Carlsbad, CA). Cells were then plated at 3 × 10^4^ cells/well of 12-well, tissue culture-treated plates (ref. no.: 25-106; Genesee Scientific, El Cajon, CA). After 24 h (or until cells reached ∼80% confluency), the cells were serum starved for 4 h in DMEM supplemented with 0.25% FBS, 10,000 U of penicillin-streptomycin, 1% L-alanyl-L-glutamine dipeptide, and 2% BSA (ref. no.: 30-AB79, lot no.: A23051103; Fitzgerald Industries International, North Acton, MA). The starvation medium was sonicated briefly to ensure proper BSA solubilization and vacuum-filtered through a 0.22 μM filter (ref. no.: 10040-436; VWR, Radnor, PA). Molecules of interest were then added to starvation media in separate conical tubes. Proper additions of DMSO and/or ethanol were made to ensure that all conditions had the same final amount of DMSO (0.1%–0.3% by v) and ethanol (0.15% by v) per well. These preparations were then sonicated at 60°C for 15 min to promote FA conjugation in the BSA-containing media before being kept in a bead bath at 37°C prior to adding to cell culture wells. Each experimental paradigm was completed at least two independent times, with a minimum of three replicates per independent experiment. At the end of the experiments, plates were manually agitated before media collection. Residual media were aspirated, and both the media and the culture plates (only containing the cells at this stage) were stored at −80°C until processing.

### PPAR target gene expression studies

#### RNA extraction and complementary DNA synthesis

RNA was extracted directly from cell culture plates using the RNeasy 96 kit (ref. no.: 74812; Qiagen, Germantown, MD) according to the manufacturer’s instructions. RNA quantity and purity were assessed using a NanoDrop (ThermoFisher Scientific). Varying amounts of RNA ranging from 200 ng to 1 μg were then used to synthesize complementary DNA (cDNA) with the High-Capacity cDNA Reverse Transcription Kit (ref. no.: 4368813; Applied Biosystems, ThermoFisher Scientific), following the manufacturer's protocol.

#### Quantitative PCR gene expression

Gene expression analysis was conducted using quantitative PCR (qPCR) on a QuantStudio 6 Flex (Applied Biosystems, ThermoFisher Scientific). Samples were loaded onto 384-well plates in triplicate, with 2–5 ng of cDNA per well, before being left at room temperature for ∼24 h to dry. Fast SYBR (ref. no.: A25743; ThermoFisher Scientific) was used for detection according to the manufacturer’s instructions, along with oligonucleotides listed in supplemental Table S5, with a final working volume of 10 μl per well. Plates were centrifuged briefly before loading into the instrument. To ensure experimental efficiency, accurate amplification, and the absence of contamination, a six-point dilution series of pooled cDNA and blank wells were included on all qPCR plates. 18S and *RPLP0* served as reference genes; Ct values were stable across samples and unaffected by FA treatments (±1 cycle, ∼0.1–0.2 Ct intrasample variation), validating ΔΔCt normalization.

### Statistical analyses

All statistical analyses were performed in R, version 4.4.2. Continuous variables were compared using Student’s *t*-tests or one-way ANOVA when assumptions of normality and homogeneity of variance were met; otherwise, nonparametric Wilcoxon-Mann-Whitney or Kruskal-Wallis tests were applied. Post hoc pairwise comparisons were assessed using Tukey’s or Dunn’s tests as applicable. Categorical variables were compared using Chi-square tests. Pairwise permutational multivariate analysis of variance tests were performed on PCA scores (PC1 and PC2) to assess differences in centroids between protein structures. Pearson’s correlation was used when data were normally distributed and showed linear relationships; Spearman’s rank correlation was applied otherwise. To compare EC_50_ and E_max_ values across ligands from the in vitro binding assays, we used compParm() from the drc package, which performs pairwise comparisons of parameter estimates from fitted dose-response models using Wald tests. For cluster analyses, k-means clustering was performed on the 3D coordinates (X, Y, and Z) of the ligand’s oxygen positions. The optimal number of clusters was determined using the elbow method by identifying an inflection point in the WSS curve. Clustering quality was further evaluated using silhouette scores computed with silhouette() from the cluster package. All statistical comparisons and visualizations of residue interaction patterns across clusters were based on these cluster assignments.

## Results

### CpFAs are fully saturated lipids with MUFA-like conformations

The presence of the three-carbon ring in the structures of CpFAs introduces local constraints to their chemical backbones, thus impacting overall three-dimensional structures. Understanding the consequences of these constraints on lipid conformation is crucial, as they may influence how these molecules behave in the context of lipid-receptor interactions and other bioactivities.

To explore this, we systematically generated a comprehensive library of known and theoretical lipids, including all possible *cis*-CpFAs, *cis*-MUFAs, and SFAs with chain lengths ranging from 12 to 24 carbons. In this paradigm, CpFAs include a single cyclopropane ring positioned from C_2_ to C_23_, and all MUFAs present a double bond at the same locations. This effort resulted in a library of 429 unique FAs, detailed in supplemental Table S2. Interestingly, only 15 of 208 generated CpFAs (7%) have been previously described (existing PubChem ID), whereas 140 of 208 predicted MUFAs (67%) and 100% of the predicted SFAs possess a PubChem ID (supplemental Table S2). To explore all potential three-dimensional conformations, for each of the 429 unique FAs, we generated up to 999 distinct conformers and measured three key structural parameters: overall end-to-end distance (Euclidean distance between the two end carbons in each conformer), compactness (the radius of gyration, which integrates the distance between each carbon and the molecule’s center of mass), and the convex hull area (which outlines and measures the molecule’s outer boundary to reflect its conformational diversity). This method allowed us to capture each lipid’s folding pattern along the range of its conformational states. A PCA was used to reduce the three-dimensional coordinates of each carbon atom in every conformer of each lipid (n = 8,170,364 atoms in total) to a two-dimensional space, allowing us to visualize the various conformations that lipids can adopt.

**Fig. 1:**
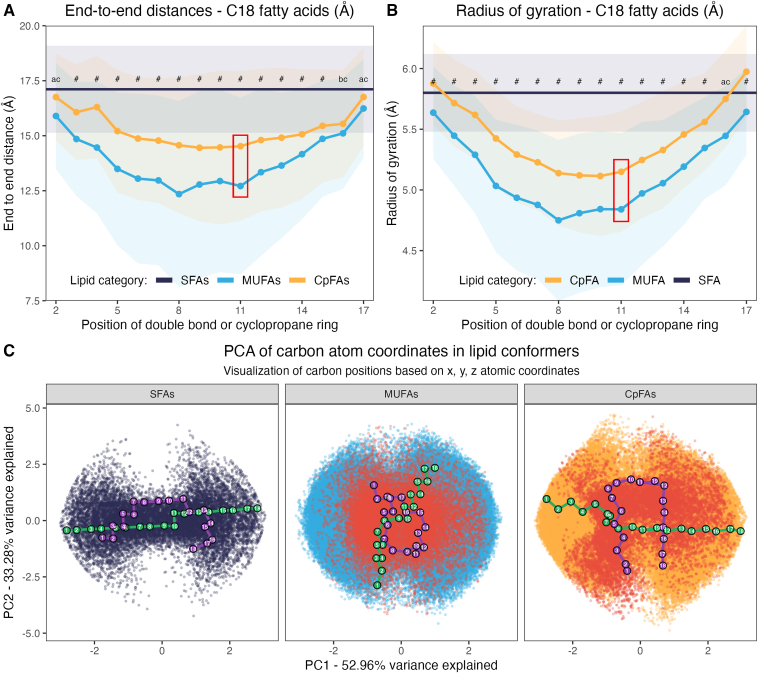
Bendability and conformational diversity of CpFAs, MUFAs, and SFAs—focus on C18 lipids. (A) End-to-end distances and (B) radius of gyration (both expressed in Å) for C18 lipids according to the position of the double bond for MUFAs (in blue) and the cyclopropane ring for CpFAs (in orange). Data are expressed as mean end-to-end distances ± standard deviation (shaded areas). The average and standard deviation value for stearic acid (in black) was projected across the plot as a reference point. The red rectangles highlight lactobacillic (C18Δ1 *cis*-11) and *cis*-vaccenic acids as illustration. Two-way ANOVA and Tukey’s honestly significant difference post hoc tests were used to compare values across lipid types (CpFA, MUFA, and SFA) and position of double bond/unsaturation (2–17); letters denote significant differences (q < 0.05) between groups, with shared "a" for CpFA versus MUFA, "b" for CpFA versus SFA, and "c" for MUFA versus SFA. # indicates that all pairwise comparisons were significant. C: PCA plots expressing all the potential locations each conformer’s carbons can adopt, according to lipid classes. Red dots represent specifically lactobacillic acid (C18Δ1 *cis*-11). Lipids projected on each PCA plot represent those with the lowest (in purple) and the highest (in green) end-to-end distances.

For illustration, typical results from these analyses using an 18-carbon chain length are depicted in Figure 1A, B, with results from lactobacillic acid (C18Δ1 *cis*-11) and *cis*-vaccenic acid highlighted in the red boxes. Similar representations for all other lipid lengths are presented in supplemental Fig. S2. These results demonstrate that both CpFAs and MUFAs can adopt significantly more “bent” conformations than SFAs. For instance, CpFAs and MUFAs display lower end-to-end distances and radii of gyrations than SFAs, across all lipid lengths (Fig. 1A and supplemental Fig. S2A). Interestingly, the extent of bending strongly depends on the location of the cyclopropane ring or double bond: for example, CpFAs and MUFAs harboring the ring or double bond near the middle section of their structure adopt more folded conformations than those with the ring or double bond located closer to either end of the molecule (Fig. 1A and supplemental Fig. S2A). The same conclusions can be drawn from the radius of gyration (Fig. 1B and supplemental Fig. S2B), demonstrating a greater potential for CpFAs and MUFAs to be more compact as compared with SFAs.

Interestingly, while both CpFAs and MUFAs have substantially more structural flexibility than SFAs, CpFA structures are somewhat less flexible when compared with MUFAs. The latter is likely because of the presence of the cyclopropane ring that, by its 2-carbon base, introduces a rigidity zone in CpFA structures. Convex hull analysis further reinforced the distinctions, demonstrating that CpFAs exhibit even greater conformational diversity than MUFAs within their pre-ring portion (supplemental Fig. S2C). This higher variability suggests that the local constraints introduced by the ring promote a different structural potential as compared with MUFAs, especially for the tails of the molecules.

Another approach to visualize conformational diversity is to project all possible locations of carbon atoms when projected on a two-dimensional PCA space (with PC1 and PC2 capturing 86% of the total variance). We observed that the carbons in SFAs are confined to a smaller, hourglass-shaped area, whereas those of MUFAs and CpFAs can span broader regions (Fig. 1C). Using lactobacillic acid for illustration, we tracked its specific folding patterns and those of its related MUFA and SFA precursors, *cis*-vaccenic and stearic acids. The carbons of all conformers for *cis*-vaccenic acid and lactobacillic acid are depicted as red dots in Fig. 1C (middle and right panels, respectively). These results clearly demonstrate the greater conformational space occupied by the MUFA and CpFA as compared with stearic acid. Furthermore, we overlaid on each PCA the two conformers for each lipid with the most extreme end-to-end distances. Lipids with the shortest end-to-end distances are shown in purple, whereas those with the longest are in green (Fig. 1C). This visualization highlights the considerable differences in conformations that these lipids can adopt.

### CpFAs, MUFAs, and SFAs are predicted to bind human PPARs

The unique structural features outlined above suggest that CpFAs may engage with PPAR receptors differently than the more rigid SFAs, in a way more akin to MUFAs. To test whether these differences influence receptor binding, we conducted an in silico docking study of all 429 known and predicted FAs across multiple structures for each human PPAR subunit (PPARα, PPARδ, and PPARγ). We selected a set of 12 high-resolution (≤2 Å) crystal structures from the Protein Data Bank and three AlphaFold-predicted 3D structures of unbound PPARα, PPARδ, and PPARγ, providing “reference” conformations unaffected by ligand binding. This approach enabled us to capture a diverse panel of protein conformations for the docking studies. Details about all 15 structures are provided in supplemental Table S1.

First, to assess structural variability within each receptor subtype and between crystal and artificial intelligence-generated structures, we performed protein alignments across all selected structures (supplemental Fig. S3A–C). The results demonstrate strong overlaps, with RMSD values generally ∼1 Å for all PPARs, indicating relative structural stability. These findings highlight the structural consistency within the different structures included in this study.

Estimated binding free energy (ΔG, expressed in kilocalories per·mole) was calculated for each of the 429 lipids and the 15 PPAR structures using the MM-GBSA method, which combines molecular mechanics and solvation (see the Materials and Methods section for details). Before delving into binding patterns across the panel of lipids, we first explored the impact of receptor structure on overall docking values. PCA plots of all ΔG values were generated and grouped by receptor structures (supplemental Fig. S3D–F). For PPARα, experimental structures (6LX8, 6KB3, 6LXA, and 7BQ0) clustered tightly, showing stable BE profiles and similar behaviors (supplemental Fig. S3D). However, the AlphaFold structure was distinctly separated from the others (permutational multivariate analysis of variance distance-based *R*^2^ >50%, *P* < 0.001; supplemental Fig. S3D), indicating that the ligand-induced conformational changes captured during crystallization had a significant impact on the binding models. PPARδ showed moderate variability with some partial overlap between the different tested structures, whereas PPARγ exhibited the most dispersion, particularly with the spread of 6D8X and the offset AlphaFold model (supplemental Table S3B, C). These results demonstrate that receptor structure directly influences lipid binding variability, with PPARα being more consistent and PPARγ the most variable.

**Fig. 2:**
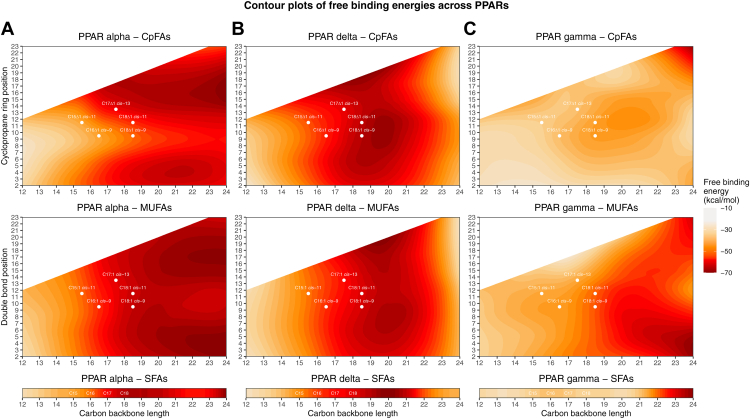
Contour plots of free BEs of 429 known and theoretical FA lipids to human PPAR α, δ, and γ. Contour plots displaying free BEs (MM-GBSA, kilocalories·per mole) of 429 lipids to PPARα (A), PPARδ (B), and PPARγ (C). The x-axis shows lipid backbone length, and the y-axis indicates double bond or cyclopropane ring position. Colors (red to yellow) represent BE, with higher values (yellow to orange) indicating weaker binding potential and more negative values (red) indicating stronger binding potential. The positions of the 14 lipids used for in vitro experiments are presented in white.

Since the ΔG values were influenced by PPAR structural variations, we averaged the structures within each PPAR isoform to derive a single ΔG value per lipid for each PPAR. As shown in the contour plots in Figure 2, virtually all FAs (SFAs, MUFAs, and CpFAs) exhibited negative binding free energies, indicating that they all fit within the binding pocket of PPARs, albeit with significant variability in terms of binding strength. Indeed, ΔG ranged from −13 to −65 kcal mol^-1^ (supplemental Fig. S4 showcasing nonmodeled individual values per lipid), which indicates widely different binding potential across FA structures. Overall, these results revealed that the predicted binding potential of all lipids was generally stronger (more negative ΔG) toward PPARα and PPARδ as compared with PPARγ. Both PPARα and PPARδ displayed a preference for FAs with chain lengths of 16 or greater carbons (Fig. 2A, B); PPARδ displayed reduced affinity for lipids exceeding 22 carbons in length (Fig. 2B).

The positions of the unsaturation or cyclopropane ring also appear to have distinct effects on binding affinity. Specifically, CpFAs showed higher docking scores when the cyclopropane ring was positioned distally along the lipid backbone in PPARα, whereas for MUFAs, either proximally or distally located unsaturations were preferred, with slightly lower scores for midpositioned unsaturation (Fig. 2A). This pattern was not observed for PPARδ (Fig. 2B) or for MUFAs and PPARγ (Fig. 2C). These findings demonstrate that chain length and the location of structural modifications (cyclopropane ring or unsaturation) can have a major influence on docking outcomes, and these effects manifest differently across PPAR isoforms. Importantly, the data also suggest that CpFAs and MUFAs exhibit distinct patterns, raising the potential for CpFA and MUFA differences in terms of interaction modalities with PPARs.

### Lipid characteristics and their contributions to PPAR BE

To further understand the factors driving binding differences between CpFAs, MUFAs, and SFAs, we analyzed the impact of lipid features (length, position of the double bond/cyclopropane ring, and length of the lipid tail) and biochemical features toward individual BEs. PCA scores plots were generated using the 429 known and theoretical FAs’ averaged ΔG values for each human PPAR isoform and evaluated the contributions of BE parameters (summed to yield the overall MM-GBSA values) and lipid structural characteristics to the projections (supplemental Fig. S5).

Across all PPAR subunits, lipophilic BE consistently emerged as an important factor, counterbalanced by solvation BE, which penalizes highly hydrophobic interactions (unless mitigated by favorable solvation effects). Coulombic BE, hydrogen bond BE, chain length, and vdW (vdW) BEs also significantly contributed to the variance separating individual FA species. This was true across all human PPAR isoforms, emphasizing the importance of polar interactions and structural features in lipid binding (emerging from either the double bond or the carboxylic group). For PPARα, the first component (PC1) is driven by a combination of lipophilic (positive) and covalent (negative) BEs, with chain length and vdW BEs playing important supporting roles in stabilizing the interaction, whereas solvation BE strongly influences the second component dimension (PC2). PPARδ FA binding exhibits a strong influence of coulombic and hydrogen bond BEs along the PC1 dimension, reflecting the importance of polar interactions, with lipophilic and vdW BEs also contributing toward the opposite, indicating a balance between hydrophobicity and polarity. Solvation BE dominates PC2 toward the negative dimension, further underscoring the impact of solvent interactions in determining binding efficiency. Finally, for PPARγ, lipophilic BE contributes toward the PC1 dimension variance, illustrating the subunit’s preference for more hydrophobic lipids, whereas solvation BE is an important factor for the PC2 dimension, highlighting the challenge of excessive hydrophobicity without proper polar compensations. Coulombic and vdW BEs further suggest that ligands require a balance between hydrophobic and polar interactions to properly interact with PPARγ, just as observed for PPARδ.

Overall, these computational insights highlight how lipid characteristics, particularly lipophilic and polar features, chain length, and positions of cyclopropane or double bonds, differentially influence binding preferences and complex interactions across PPAR isoforms.

### TR-FRET assays revealed different binding preferences across PPAR subunits

To experimentally validate the computational modeling indicating binding of CpFAs to PPARs, we selected five CpFAs commercially available or custom synthesized (C15Δ1 *cis*-11, C16Δ1 *cis*-9, C17Δ1 *cis*-13, C18Δ1 *cis*-9, and C18Δ1 *cis*-11), their five MUFA precursors (C15:1 *cis*-11, C16:1 *cis*-9, C17:1 *cis*-13, C18:1 *cis*-9, and C18:1 *cis*-11), and their four saturated equivalents (pentadecanoic, hexadecenoic, heptadecanoic, and octadecanoic acids). The binding potential of these 14 lipids on isolated binding domains of human PPARα, PPARδ, and PPARγ was measured using LanthaScreen TR-FRET assays. These coactivator assays measure ligand-induced receptor activation by detecting energy transfer between a terbium-labeled antibody and a fluorescent coactivator peptide, which binds the LBD only upon ligand-induced conformational changes.

**Fig. 3:**
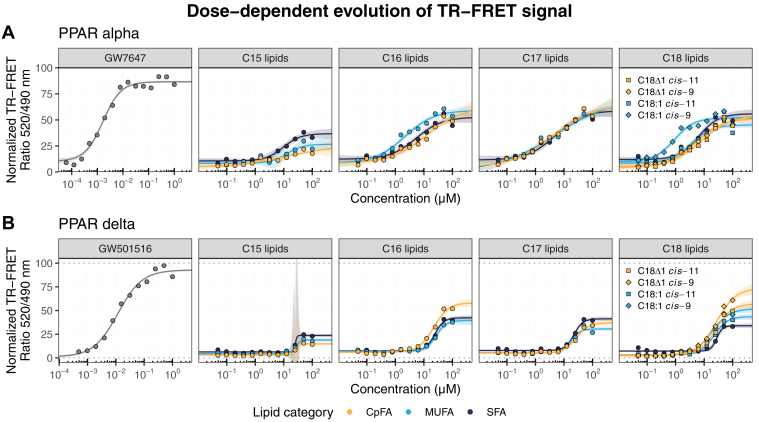
In vitro binding of FAs to human PPAR isoforms using TR-FRET binding assays. A and B: Dose-dependent evolution of TR-FRET signal upon ligand binding for PPARα and PPARδ, respectively.

The TR-FRET assay results presented in Table 1 and Figure 3 revealed distinct binding and activation profiles across the three human PPAR subunits. First, for PPARα, the EC_50_ values for FAs ranged from 1 to 10 μM (Table 1). Lipids with a 15-carbon backbone had a modest binding to PPARα: for example, EC_50_ of ∼10–14 μM, and at 100 μM, they produced about 20%–30% (E_max_) of the response observed with the highest concentration of GW7647, a potent PPARα synthetic agonist. This is compared with longer-chain FAs that induced a higher E_max_ of approximately 50%–65% of that induced by GW7647 and with EC_50_ values typically between ∼1 and 12 μM. C17:1 *cis*-13 was the lipid with the highest E_max_ at 70%, followed by C17Δ1 *cis*-13 with an E_max_ of 65%. Overall, these results support the in silico observations that binding patterns of FAs to human PPARα are generally more robust and consistent for compounds longer than 15 carbons regardless of lipid class (Fig. 2).

**Table 1:** Coactivator recruitment characteristics of select CpFAs, MUFAs, and SFAs on human PPARα and PPARδ LBDs using TR-FRET coactivator recruitment assays

Ligand/fatty acid		PPARα		PPARδ	
		EC_50_ (μM)	E_max_ (%)	EC_50_ (μM)	E_max_ (%)
Agonists					
GW7647		1.65 (1.6, 1.7)	86.57 (85.5, 87.6)		
GW501516				11.83 (11.2, 12.4)	92.8 (91.8, 93.8)
FAs					
CpFA	C18Δ1 cis-11	7.65 (5, 10.3) a	54.7 (48.8, 60.6) a	25.81 (22, 29.6) ab	57.03 (52.5, 61.6) a
MUFA	C18:1 cis-11	3.5 (2.5, 4.5) b	45.22 (42.4, 48) b	22.02 (18.9, 25.2) ac	43.47 (40.1, 46.9) c
CpFA	C18Δ1 cis-9	5.23 (3.8, 6.7) ab	51.38 (47.3, 55.5) ab	27.1 (21.3, 32.9) ab	73.56 (65.9, 81.2) b
MUFA	C18:1 cis-9	0.91 (0.7, 1.1) c	51.98 (49.8, 54.1) a	18.53 (15.4, 21.7) c	51.91 (47.7, 56.1) a
SFA	C18	6.37 (4.4, 8.3) ab	55.92 (51.5, 60.4) a	27.39 (24.8, 30) b	33.97 (31.7, 36.2) d
CpFA	C17Δ1 cis-13	4.12 (1.3, 6.9) a	65.01 (52.5, 77.5) a	26.77 (21.5, 32.1) a	37.11 (33.1, 41.1) a
MUFA	C17:1 cis-13	4.7 (−1.3, 10.7) a	69.86 (43, 96.7) a	17.78 (15.2, 20.4) b	30.57 (28.7, 32.5) b
SFA	C17	5.04 (2.8, 7.3) a	58.93 (52.5, 65.4) a	22.34 (20, 24.7) a	41.26 (39, 43.5) a
CpFA	C16Δ1 cis-9	12.18 (0.7, 23.7) ab	62.28 (45.2, 79.4) a	21.96 (18.9, 25) a	58.09 (53.9, 62.3) a
MUFA	C16:1 cis-9	2.13 (1.2, 3) a	59.11 (53.7, 64.5) a	26.42 (20.6, 32.2) a	39.46 (34.8, 44.1) b
SFA	C16	5.43 (2.9, 8) b	52.61 (46.8, 58.5) a	24.11 (21.4, 26.8) a	42.3 (39.9, 44.7) b
CpFA	C15Δ1 cis-11	14.1 (-57.4, 85.6) a	26.26 (−3.2, 55.8) ab	25.58 (18.3, 32.8) a	14.87 (13.7, 16) a
MUFA	C15:1 cis-11	12.78 (5.1, 20.4) a	26.64 (18.8, 34.5) a	25.67 (21.8, 29.6) a	18.9 (16.9, 20.9) b
SFA	C15	9.6 (5, 14.2) a	36.78 (31.6, 41.9) b	25.57 (12.7, 38.4) a	23.71 (22.1, 25.3) c

EC_50_, half maximal effective concentration (in μM); E_max_, maximum efficacy (% relative to PPAR isoform-specific agonist controls)

95% confidence intervals are presented in parentheses. Statistics were evaluated within FA chain lengths using the comParm() function from the drc package. Lipids sharing a letter within a given chain length are not significantly different; those not sharing a letter differ significantly.

The observations made for PPARα regarding lipids with 15-carbon backbones were also true for PPARδ, and longer FAs produced notably different responses depending on the specific molecule. For example, the CpFA dihydrosterculic acid (C18Δ1 *cis*-9) reached a maximum response (E_max_) close to 74% of the TR-FRET ratio induced by the highest concentration of the agonist GW501516 (Table 1), a value higher than that of the C18 CpFA lactobacillic acid (57%). Furthermore, CpFAs with chain lengths of 16 and 18 outperformed both their related MUFAs and SFAs, as seen with higher maximum effects and no confidence-interval overlaps (Fig. 3B). This was particularly clear in the C16–C18 range, where CpFAs consistently showed higher E_max_ values (58%–74%) than MUFAs (39%–52%) and SFAs (34%–42%). Interestingly, the EC_50_ values observed for PPARδ were generally higher (15–30 μM) and more uniform than those observed for PPARα, suggesting that the variability in response is primarily driven by efficacy rather than potency.

The PPARγ binding assay was more challenging to interpret (supplemental Fig. S6). Although rosiglitazone (PPARγ agonist) produced the expected dose-dependent increase in the 495/520 nm signal ratio (but with a lower dynamic range), experiments with CpFAs and other FAs (including those established as ligands of PPARγ) showed a stable 495 nm signal but inconsistent and sometimes sharply reduced 520 nm signals, a phenomenon not observed for PPARα or PPARδ (supplemental Figs. S7 and S9). We speculate that these patterns are indicative of complex binding dynamics of FAs with PPARγ, leading to as-yet-to-be-defined differences in LBD-coactivator peptide recruitment or fluorescence quenching when compared with PPARα and PPARδ. Therefore, calculation of EC_50_ or related measures was not possible with this assay.

To understand the reliability of the in silico modeling results, we correlated predicted binding affinities (ΔG values from Fig. 2) with the fitted maximum effect and EC_50_ values obtained in vitro with the TR-FRET assay for PPARα and PPARδ. The E_max_ values show a significant negative correlation with ΔG values for both PPARα and PPARδ, with Pearson’s *r* of −0.56 and −0.71, respectively (supplemental Fig. S10A, B), indicating that lipids predicted to bind more tightly from the in silico models generally produced stronger activation signals in the TR-FRET assays. By contrast, EC_50_ values were only positively correlated with ΔG for PPARα and not for PPARδ (supplemental Fig. S10B, C). This further suggests that, in the case of PPARδ, the strength of lipid-induced activation is more closely tied to the quality of binding (maximum effect, E_max_) than its potency (EC_50_). These results support the idea that while computational docking results can reliably predict ligand-receptor interactions, the downstream biological response can be shaped by PPAR isoform-specific dynamics and FA structures.

Overall, the findings for PPARα and PPARδ binding assays validate the computational predictions that CpFAs can bind human PPAR isoforms. The data further emphasize that lipid characteristics such as chain length and unsaturation or cyclopropane ring position can significantly impact binding potential.

### Lipid orientations within PPAR LBDs shape distinct interaction profiles

To better understand the nuances in PPARs’ interactions with lipids observed both in vitro and in silico, we examined the positions lipids can adopt within the LBDs of all three PPARs and how their orientation might influence interactions with specific amino acid residues. To do so, 100 conformers for each of the 14 lipids tested in the TR-FRET experiments were used in docking models. Only poses that successfully converged during energy minimization were used to ensure that physically realistic and well-optimized structures were evaluated. Since the final number of retained poses varied across lipids, we subsampled each set to include an equal number of poses per FA. The extracted atomic coordinates for each lipid were used to plot the positions of the carboxylic acid oxygen atoms. To simplify visualization, we averaged the coordinates of both oxygen atoms in each lipid, representing them as a single dot.

**Fig. 4:**
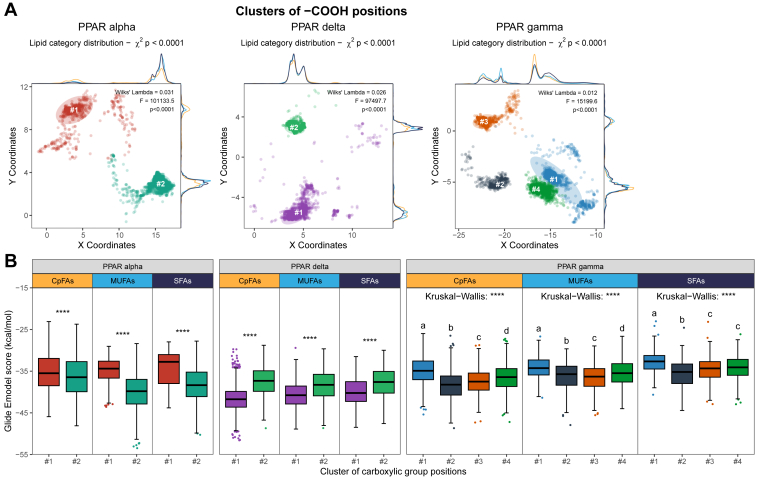
FAs can adopt multiple positions within the LBD of all subunits of human PPARs, which impacts the predicted affinity. A: Two-dimensional positions of the carboxyl group of all conformers generated for the 14 lipids tested in vitro (supplemental Table S3) within the LBDs of human PPARα, PPARδ, and PPARγ, respectively. Each conformer was grouped into spatial clusters using k-means clustering. Density curves along the top and right margins of each plot indicate the distribution of lipid categories (CpFA, MUFA, and SFA) within the coordinate space. Lipid enrichment across clusters was assessed by Chi-squared tests, and intercluster separation was evaluated via multivariate ANOVA. B: Comparison of average glide E_model_ scores (kilocalories·per mole) across positional clusters within each PPAR LBD, stratified by lipid category. For PPARα and PPARδ, comparisons were performed using the Mann-Whitney test; for PPARγ, Kruskal-Wallis test followed by Dunn’s post hoc comparisons was used. Boxplots sharing the same letter are not significantly different; differing letters indicate statistically significant differences.

Using k-means clustering, distinct clusters of lipid carboxyl-group positions within the LBDs of human PPARα, PPARδ, and PPARγ were identified. As shown in Figure 4A, two dominant orientations emerged for PPARα and PPARδ and four orientations for PPARγ. The distribution of CpFAs among clusters differed from that of MUFAs and SFAs (supplemental Fig. S11A, B). For instance, in PPARα, 32% of all CpFA conformers fell into cluster #1 compared with just 11% and 7% for MUFAs and SFAs, respectively. In PPARδ, 60% of CpFAs aligned with cluster #1 versus 45% for MUFAs and 39% for SFAs. These results suggest that CpFAs are more likely to adopt different positions within the LBD. In contrast, for PPARγ, although four carboxylic location clusters were identified, the lipid class proportions were more consistent across clusters, except for a higher density of MUFAs oriented in cluster #2 and of SFAs in cluster #1 (supplemental Fig. S11C).

To assess the impact of lipid orientation on binding potential, we analyzed the average Glide Emodel scores (expressed in kilocalories per·mole) by lipid class and cluster (Fig. 4B). Glide Emodel integrates both the docking score and the ligand’s internal strain energy, making it particularly useful for comparing how different conformers or binding positions influence ligand-receptor interactions. For PPARα, CpFAs’ clusters had minimal effect on their BE, whereas both SFAs and MUFAs showed reduced docking scores when positioned in cluster #2 (Fig. 4B). This suggests that the tendency of CpFAs to adopt a cluster #1 orientation does not confer a binding advantage. This also suggests that their effect on PPARα might be similar regardless of orientation, which seems not to be the case for MUFAs and SFAs (Fig. 4B). For PPARδ, all lipid classes exhibited lower docking scores when oriented in cluster #1, indicating that the increased prevalence of CpFAs in this cluster may provide a favorable binding configuration. In PPARγ, while docking scores varied significantly between clusters, the differences were modest. Intriguingly, cluster #2 orientation appeared to be slightly preferred, despite representing a minor orientation within the LBD.

To better understand whether these orientation differences translated into distinct molecular contacts, we next examined lipid-protein interactions at the residue level. To do so, we generated residue interaction fingerprints for each conformer-PPAR pair, aggregating all interaction types (supplemental Figs. S12 and S13), enabling a direct comparison of how structural differences translate into specific molecular engagements across isoforms.

First, when comparing fingerprints across lipid classes (CpFAs, MUFAs, and SFAs), we observed no major differences; in fact, the interaction patterns were highly similar across all PPARs (supplemental Fig. S12). Despite sequence variability among PPAR isoforms, this confirms a high degree of structural conservation within their LBDs. In PPARα, the residues most engaged across all lipids included, for example, Cys276, Ser280, Tyr314, Val332, His440, and Tyr464. In PPARδ, commonly engaged residues included, for example, Phe246, Cys249, Thr253, Leu294, Asn307, and Tyr437. In PPARγ, interaction patterns were dominated by contacts in Phe292, Cys313, Arg316, Leu358, Ile369, and Ser370.

While residue-level fingerprints did not differ substantially across FA categories, major differences emerged when lipids were grouped by clusters of positions (supplemental Fig. S13). For PPARα, multiple ligand interactions with Cys276 and Ser280 were preserved in both clusters (supplemental Fig. S14A). However, lipids in cluster #1 showed more contact with Asn219, Met220, Thr279, Thr283, Ile317, and Tyr334, whereas cluster #2 lipids were more engaged with Gln277, Tyr314, Met330, Leu460, and Tyr464 (supplemental Fig. S14A). This suggests that cluster #1 interacts more with residues located earlier in the sequence, whereas cluster #2 engages with more distal residues. For PPARδ, cluster #1 lipids had greater interactions with Lys229, Ile290, Thr252, Thr256, and Asn307. In contrast, cluster #2 lipids interacted more with distal residues, such as Tyr437, Phe246, Gln250, Leu433, and Met417, echoing the same spatial contact pattern observed in PPARα (supplemental Fig. S14B). In the case of PPARγ, lipids in clusters #1, #2, and #3 only interacted modestly with distal residues, such as Leu481, His477, Leu497, and Tyr501, whereas lipids in cluster #3 had stronger interactions within this region (supplemental Fig. S14C). On the other hand, lipids in clusters #2, #3, and #4 showed limited contact with proximal residues, such as Phe254, Pro255, and Leu256, which were more prominently contacted by lipids in cluster #1 (supplemental Fig. S14C). These patterns therefore suggest that cluster #1 is more prone to contacts with the N-terminal portion of the receptors, cluster #3 more toward the C-terminal region, whereas clusters #2 and #4 are more centered around the core of the LBD.

Taken together, these results indicate that while CpFAs, MUFAs, and SFAs share a conserved set of interacting residues within each PPAR isoform, their positioning within the LBD can induce strongly divergent interaction patterns. These orientation-dependent differences may influence downstream signaling by altering the mode of PPAR engagement.

### CpFAs modulate PPAR-target gene expression in 3T3-L1 cells

Having confirmed through in silico modeling and in vitro biochemical assays that CpFAs bind to the LBDs of human PPAR isoforms, we next sought to validate the functional consequences of CpFA-mediated PPAR activation on target gene expression in a cell model. Given their extensive use in the literature, their expression of all PPAR subunits, and their established role in lipid metabolism, we selected 3T3-L1 murine preadipocytes as a model. Angiopoietin-like 4 (Angptl4) transcript measurement was selected since this gene is a well-known, potent, and sensitive marker of pan-PPAR activation (31).

We first treated 3T3-L1 cells with the same PPAR agonists used for TR-FRET binding assays (GW7647 for PPARα, GW501516 for PPARδ, and rosiglitazone for PPARγ—see supplemental Table S4) and confirmed increased *Angptl4* expression by these agonists. The 24-h treatments led to dose-dependent inductions of *Angptl4* expression (supplemental Fig. S15). Significant upregulation was observed at 10 μM for all three agonists, confirming that *Angptl4* is transcriptionally upregulated by the activation of all PPAR subunits. Fold changes as compared with the vehicle were high, averaging at 11, 23, and 5 for PPARα, PPARδ, and PPARγ, respectively (supplemental Fig. S15).

**Fig. 5:**
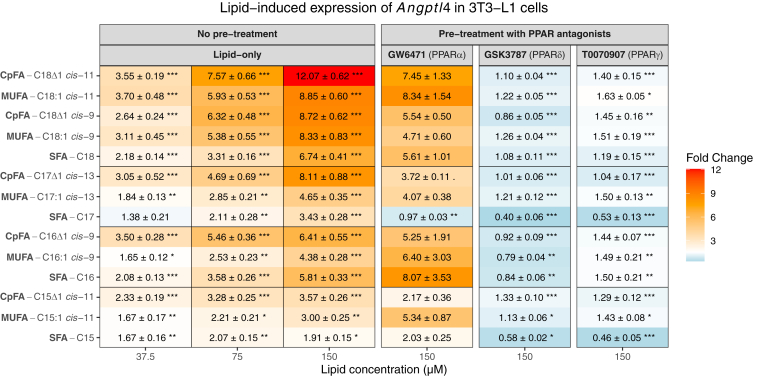
mRNA expression of the pan-PPAR target gene *Angptl-4* in 3T3-L1 murine preadipocyte cells, without and with PPAR-specific chemical antagonists. The left side of the figure represents the fold-change induction of *Angptl-4* expression after 24 h of treatment with increasing concentrations of SFAs, MUFAs, or CpFAs. All values were tested for significance (Kruskal-Wallis test followed by a Dunn’s post hoc test with Holm correction) against the fold-change values of the vehicle condition. On the right-hand side is displayed the induction of *Angptl-4* expression after treatment with the same lipids but with a concurrent treatment with 10 μM PPAR isoform-specific antagonists, namely GW6471 for PPARα, GSK3787 for PPARδ, and T0070907 for PPARγ. All values were tested for significance using the same test as before but against the values of the nonantagonized lipid at 150 μM. All *P* values were further adjusted to control for multiple testing (Bonferroni). q < 0.1, ∗q < 0.05, ∗∗q < 0.01, and ∗∗∗q < 0.001.

Having confirmed *Angptl4* as a reliable transcriptional readout of PPAR activation, we next assessed whether the 14 lipids of interest that were used in biochemical binding assays could induce its expression. As shown in the left panel of Figure 5, all CpFAs, MUFAs, and SFAs induced a significant upregulation of *Angptl4* in a dose-dependent manner as compared with vehicle controls. Importantly, the magnitude of induction was generally higher for CpFAs compared with their MUFA and SFA chain-length matched counterparts. The greatest induction was observed for lactobacillic acid (C18Δ1 *cis*-11), followed closely by dihydrosterculic acid (C18Δ1 *cis*-9) and C17Δ1 *cis*-13. These findings are consistent with our in silico and biochemical binding assays, reinforcing that CpFAs function as potent PPAR ligands.

To further validate that CpFA-induced *Angptl4* expression is at least partially mediated by interaction with PPARs, 3T3-L1 cells were pretreated with selective PPAR antagonists (GW6471 for PPARα, GSK3787 for PPARδ, and T0070907 for PPARγ—see supplemental Table S4) for 4 h before exposing them to the lipids for 24 h (while keeping the antagonist present). Intriguingly, PPARα antagonism did not inhibit PPARα-mediated *Angptl4* expression, whereas PPARδ and PPARγ antagonism resulted in a reduction of the expression of *Antpgl4* by ∼50% (supplemental Fig. S16). We therefore used antagonist-alone conditions as a control for fold-change calculations. In light of the poor utility of GW6471 as a PPARα antagonist, it is perhaps not surprising that the addition of this compound with CpFAs, MUFAs, and SFAs failed to blunt FA-induced *Angptl4* expression (Fig. 5). In contrast, FA activation of target gene expression was virtually abolished by the addition of PPARδ and PPARγ antagonists (Fig. 5).

Overall, these results provide direct functional evidence that CpFAs modulate PPARδ and PPARγ activity in 3T3-L1 cells, leading to the transcriptional regulation of downstream target genes. Combined with our docking and biochemical binding studies, these findings indicate that CpFAs can act as bona fide PPAR ligands, with the acknowledgment that further studies are needed (e.g., using different cell models and alternative PPARα antagonists) in order to fully characterize CpFA activation of PPARα.

## Discussion

This study demonstrates that CpFA xenolipids exhibit unique structural and functional properties that distinguish them from SFAs and convey some resemblance to MUFAs, despite CpFAs being fully saturated. By generating an extensive virtual lipid library spanning all possible saturated, monounsaturated, and monocyclopropane lipids with chain lengths from 12 to 24 carbons, we were able to systematically analyze how chain length and bond diversity impact conformational landscapes and PPAR receptor binding potential. Some of these aspects were confirmed in vitro for a subset of lipids to evaluate their actual binding and downstream gene activation capacities. Our findings show that the presence of the cyclopropane ring induces a significant bending and compaction of the lipid structure, which translates into robust binding to PPARs, including PPARα and PPARδ. These results extend previous observations on the CpFA lactobacillic acid as a PPARγ ligand (29) and provide novel evidence that CpFAs (including newly described odd-chain CpFAs) interact functionally with PPARα and PPARδ. These observations provide new insights into the potential physiological roles of CpFAs emanating from foods and gut microbiota.

The notion that the level of unsaturation of a lipid impacts its overall flexibility is widely accepted from a physical standpoint but has mostly been observed indirectly (32, 33, 34). Indeed, most of the work done so far focused on membrane flexibility, linking saturated lipids to stiffer, more ordered membranes, while the presence of unsaturated lipids increases their fluidity and dynamics (35, 36). Our in silico results demonstrate that unlike SFAs, CpFAs can “bend” (as seen by lower end-to-end distances and radius of gyration), which allows them to adopt more compact structures akin to MUFAs. These observations align with previous studies demonstrating that while CpFAs tend to slightly decrease membrane fluidity (as compared with *trans* FAs), their effect is closer to that of unsaturated FAs rather than the pronounced stiffening seen with *trans*- or SFAs (33, 35, 37, 38). However, the impact of CpFAs’ unusual conformations was never explored in the context of ligand-receptor interaction.

MUFAs, and to a lesser extent SFAs, are well-known natural ligands of PPAR receptors, with varying potencies (39, 40). The described effects of those two classes of lipids on PPARs are greatly variable between studies and methodologies but tend to demonstrate that some MUFAs, such as oleic and palmitoleic acids, can bind all PPARs in concentrations in the micromolar range (41). The effects of SFA are more limited, although they have shown some weak binding affinity toward PPARα and PPARγ (41, 42). These examples illustrate that one cannot assume that all FAs are alike with respect to binding dynamics and avidity (even if chain length is shared), especially when considering disparate PPAR isoforms where binding and functional activities of any given FA can differ. To date, only one study has explored the binding potential of a CpFA in silico and in vitro, assessing lactobacillic acid (C18Δ1 *cis*-11) binding to PPARγ (29). Lactobacillic acid was the lipid with the lowest reported K_d_ value (∼100 μM) among nine lipids tested in vitro (29), with stronger binding than that of polyunsaturated FAs like eicosapentaenoic acid, previously reported as a potent ligand of PPARγ (41). Herein, we tested the hypothesis that CpFAs can also serve as novel ligands of human PPARα and PPARδ, and we tested, for the first time, the ability of several structurally diverse CpFAs to bind and activate human PPARs. Our in silico results demonstrate that all lipids, regardless of class, fit within PPARs’ binding pockets, but with highly varying degrees of strength (ΔG values) related to specific structural features like FA chain length or position of the cyclopropane ring/unsaturation. This observation is consistent with the large size of PPARs’ binding pockets (43, 44, 45, 46). Nevertheless, theoretically fitting within the binding pocket of a PPAR does not confirm bioactivities. Conformational dynamics, coactivator/corepressor recruitment, heterodimerization with retinoid X receptors, and post-translational modifications are some of the many parameters involved in PPAR pathways that cannot be evaluated in silico (47). This prompted us to perform in vitro work on a subset of available CpFAs.

TR-FRET coactivator recruitment assays are a simplified system with isolated LBD portions of the human PPAR receptors put in contact with labeled coactivator peptides. The assays provide more details about the conformational changes induced by ligand binding, and they generate estimations of binding affinity and intensity. The results for PPARα corroborated our in silico predictions: all tested lipids can fit within the LBD, and the TR-FRET assay demonstrated that they induced comparable conformational changes and potencies, as reflected by EC_50_ values in the ∼2–5 μM range. On the other hand, the affinity was a bit lower for PPARδ (EC_50_ values ∼20–30 μM). Interestingly, CpFAs of chain lengths of 16 and 18 outperformed their related MUFAs and SFAs in terms of maximum binding intensity. For one CpFA, the maximal ratio change reached 75% of that induced by the highest concentration of the chemical agonist, translating to very potent activation potential. Unfortunately, we were not able to make confident interpretations of the results generated with the PPARγ assay. Our hypothesis is that lipids induced an unexpectedly sharp drop in the 520 nm signal by either *i*) inducing a conformational change within the PPARγ LBD, disrupting the binding of the coactivator peptide or *ii*) interacting with the coactivator peptide itself, sequestering it. Unfortunately, trials to use other coactivator peptides were not fruitful. Importantly, previously validated PPARγ lipid ligands (such as oleic acid (41)) also exhibited the drop in 520 nm signal, which further suggests an issue with the assay itself. Overall, the results from biochemical binding studies corroborated molecular modeling studies and indicate that several well-established CpFAs (C16Δ1 *cis*-9, C18Δ1 *cis*-9, and C18Δ1 *cis*-11) and newly characterized odd-chain CpFAs (C15Δ1 *cis*-11 and C17Δ1 *cis*-13 (24)) bind the LBD of human PPARα and PPARδ isoforms.

In addition to differences in binding potencies and efficacies, in silico modeling suggested that CpFAs can adopt alternate orientations within the PPAR LBDs compared with MUFAs and SFAs; the latter tended to be more constrained, at least for PPARα and PPARδ. This broader space distribution in the ligand binding pocket of PPARs suggests greater flexibility in how CpFAs engage the receptor cavity, which is consistent with the notion that molecule flexibility might impact positioning within PPARs’ LBD (48). While all FAs engaged a conserved core set of residues, CpFAs showed a higher tendency to occupy deeper or alternative regions of the binding pocket, particularly in PPARα and PPARδ, resulting in different residue interaction fingerprints. For instance, CpFAs were more likely to adopt a cluster #1 position in PPARδ; therefore, they were more frequently in contact with residues like Asn307, Lys229, and Ile290, whereas MUFAs and SFAs were preferentially in cluster #2 positions, engaging with more distal residues (Tyr437, Leu433) near the entrance of the cavity. Such orientation shifts are not trivial: ligand position within the PPAR LBD has been shown to critically influence the degree of AF-2 helix stabilization, coactivator recruitment, and transcriptional output (43, 46, 49). Full agonists, such as synthetic thiazolidinediones, typically stabilize the AF-2 helix through direct polar interactions, whereas partial agonists, including some endogenous FAs, adopt alternate poses that favor weaker activation (46, 49). Our findings suggest that CpFAs, by favoring alternate orientations and distinct residue engagements, may promote receptor conformations associated with partial activation profiles, particularly for PPARδ. These observations emphasize a potential mechanistic basis for the differential activation strength we observed in TR-FRET assays and support the notion that lipid structural features play a decisive role in modulating nuclear receptors.

To further assess whether these differences in interactions with PPARs, particularly for CpFAs, could impact downstream PPAR signaling, we examined the expression of *Angptl4*, a direct transcriptional target of PPARs involved in lipid metabolism, angiogenesis, and energy balance (31). CpFA treatments robustly upregulated *Angptl4* expression in 3T3-L1 preadipocytes, with antagonist experiments confirming PPARδ and PPARγ activation. Unfortunately, the antagonist compound for PPARα lacked potency in this assay, and thus, future studies are needed to fully characterize the activation of PPARα by CpFAs in cell models. These results align with previous studies showing that *Angptl4* is highly inducible by glitazones (PPARγ agonists) in adipose tissues (50) and by PPARδ activation in skeletal muscle and endothelial cells (51). Although studies have shown that PPARα can regulate *Angptl4* expression in hepatic contexts (52), its influence appears less prominent in preadipocytes, consistent with our observations. Other studies have reported an FA-mediated regulation of *Angptl4*: oleic and other unsaturated FAs upregulate *Angptl4* in myocytes via PPARδ-dependent pathways (53, 54). Our results therefore support the concept that CpFAs are chemically saturated but robustly activate *Angptl4* transcription by sharing some functional behaviors of MUFAs because of structural similarities. This supports previous evidence demonstrating that ligand structure, particularly chain flexibility and bending, critically influences PPAR activation profiles (43). Thus, CpFAs represent a unique class of xenolipids capable of selectively modulating PPARα/δ/γ-driven gene programs, potentially impacting systemic lipid handling, metabolic homeostasis, and other physiological outcomes.

Our study represents the first to comprehensively evaluate fatty structural phenomena and PPAR binding characteristics across all known and theoretical FAs from the CpFA, MUFA, and SFA classes with chain lengths of 12–24. The experiments are also novel in confirming CpFAs’ binding and activation across all 3 human PPAR isoforms. The studies do have several limitations. The in silico docking and molecular dynamics simulations rely on computational models that do not fully capture the complexity of biological systems, especially in the context of nuclear receptors. Although partly corroborated by our in vitro findings, these results would benefit from direct observations through structural methods (e.g., X-ray crystallography) to define precise binding modalities. Another limitation of this work is the rather limited scope of the in vitro studies, as we tested a subset of 14 of the 429 lipids we generated in silico because of limited commercial availability of CpFAs. Nevertheless, we utilized the largest CpFA panel described to date, including two odd-chain CpFAs (24) and three even-chain CpFAs that were previously detected in human blood and adipose tissue (25, 27) and derived from the commonly consumed and stored MUFAs (55), underscoring their clinical relevance. Nevertheless, these five CpFAs do not cover the computationally predicted "best hits" for PPAR binding. Synthesizing additional CpFAs, especially those with optimal chain lengths and cyclopropane ring positions, could help validate enhanced binding properties. We acknowledge that direct binding kinetics were not assessed in this study; future work employing biophysical methods such as surface plasmon resonance to examine CpFA interactions with PPARs, especially with PPARγ, will be important to complement our docking and cell-based findings. Another limitation is the use of a single-cell culture model; while robust for assessing Angptl4 expression across many conditions, future experiments using differentiated adipocytes, gene-silencing approaches such as siRNA-mediated knockdown, and testing additional target genes across multiple cell types will be important to further validate and extend our findings. Importantly, our cell-based experiments were conducted in mouse 3T3-L1 preadipocytes. Human and murine PPAR isoforms differ in their expression, activation, and function (56, 57); therefore, additional studies in human-derived systems will be required to fully validate these findings. Furthermore, analysis of CpFA bioactivities and PPAR actions could benefit from studies focused on broader transcriptomic, proteomic, and metabolomic profiling in PPAR-responsive cells. This is particularly important for macrophages, since PPARs are implicated in regulating the immune system and dampening inflammation (58). Finally, future studies using animal models will be valuable to assess the systemic effects of dietary or microbially derived CpFAs on PPAR activity and metabolic outcomes. Clinical studies could explore whether acute or chronic CpFA exposure alters metabolic health markers in humans, particularly given their presence in the diet and their potential synthesis by gut bacteria.

In conclusion, the present work establishes CpFAs as a distinct class of SFAs with MUFA-like structural and functional properties, yet having distinct structural characteristics differentiating the CpFAs from chain length-matched MUFA and SFA counterparts. These findings challenge the broad categorization of saturated lipids as uniform in their biological effects and highlight the role of xenolipids in modulating host physiology. This offers new perspectives for understanding lipid bioactivities at the interface of microbiota, diet, and host metabolism and suggests that CpFAs could be novel modulators of health through activation of one or more of the PPAR nuclear receptors.

## Data availability

In silico data, in vitro binding data, and in vitro cell assay data generated for this study are available upon request from Dr Sean H. Adams (University of California, Davis; shadams@health.ucdavis.edu). No publicly archived datasets or software code are associated with this article.

## Supplemental data

This article contains supplemental data (59, 60, 61, 62, 63, 64, 65).

## Conflict of interest

S. H. A. is the founder and principal of XenoMed, LLC (dba XenoMet), which is focused on research and discovery in the area of microbial metabolism. XenoMed had no part in the research design, funding, results, or writing of the article. S. H. A. and J. D. are coinventors on a patent application in the area of CpFAs. All other authors declare that they have no conflicts of interest with the contents of this article.

## References

[1] Dekkers K.F., Sayols-Baixeras S., Baldanzi G., Nowak C., Hammar U., Nguyen D. (2022). An online atlas of human plasma metabolite signatures of gut microbiome composition. Nat. Commun..

[2] Deng K., Xu J., Shen L., Zhao H., Gou W., Xu F. (2023). Comparison of fecal and blood metabolome reveals inconsistent associations of the gut microbiota with cardiometabolic diseases. Nat. Commun..

[3] Wikoff W.R., Anfora A.T., Liu J., Schultz P.G., Lesley S.A., Peters E.C. (2009). Metabolomics analysis reveals large effects of gut microflora on mammalian blood metabolites. Proc. Natl. Acad. Sci. U. S. A..

[4] Lai Y., Liu C.-W., Yang Y., Hsiao Y.-C., Ru H., Lu K. (2021). High-coverage metabolomics uncovers microbiota-driven biochemical landscape of interorgan transport and gut-brain communication in mice. Nat. Commun..

[5] Folz J., Culver R.N., Morales J.M., Grembi J., Triadafilopoulos G., Relman D.A. (2023). Human metabolome variation along the upper intestinal tract. Nat. Metab..

[6] Dyall S.C., Balas L., Bazan N.G., Brenna J.T., Chiang N., da Costa Souza F. (2022). Polyunsaturated fatty acids and fatty acid-derived lipid mediators: recent advances in the understanding of their biosynthesis, structures, and functions. Prog. Lipid Res..

[7] Badawy S., Liu Y., Guo M., Liu Z., Xie C., Marawan M.A. (2023). Conjugated linoleic acid (CLA) as a functional food: is it beneficial or not?. Food Res. Int..

[8] Taormina V.M., Unger A.L., Schiksnis M.R., Torres-Gonzalez M., Kraft J. (2020). Branched-chain fatty acids—an underexplored class of dairy-derived fatty acids. Nutrients.

[9] Koh A., Molinaro A., Ståhlman M., Khan M.T., Schmidt C., Mannerås-Holm L. (2018). Microbially produced imidazole propionate impairs insulin signaling through mTORC1. Cell.

[10] Konopelski P., Mogilnicka I. (2022). Biological effects of Indole-3-Propionic acid, a gut microbiota-derived metabolite, and its precursor tryptophan in mammals’ health and disease. Int. J. Mol. Sci..

[11] Kramer D.J., Wang W., Loque I., Walters-Laird C.J., Morisseau C., Xiao X. (2025). Microbial biotherapeutic metabolite alleviates liver injury by restoring hepatic lipid metabolism through PPARα across the gut-liver axis. mBio.

[12] Hofmann K., Lucas R.A. (1950). The chemical nature of a unique fatty acid. J. Am. Chem. Soc..

[13] Chung A.E., Law J.H. (1964). Cyclopropane fatty acid synthetase: partial purification and properties. Biochemistry.

[14] Bao X., Katz S., Pollard M., Ohlrogge J. (2002). Carbocyclic fatty acids in plants: biochemical and molecular genetic characterization of cyclopropane fatty acid synthesis of Sterculia foetida. Proc. Natl. Acad. Sci. U. S. A..

[15] Eras J., Oró R., Torres M., Canela R. (2008). Direct quantitation of fatty acids present in bacteria and fungi: stability of the cyclopropane ring to chlorotrimethylsilane. J. Agric. Food Chem..

[16] Rězanka T., Vokoun J., Slavíček J., Podojil M. (1983). Determination of fatty acids in algae by capillary gas chromatography—mass spectrometry. J. Chromatogr. A.

[17] Chang Y.Y., Cronan J.E. (1999). Membrane cyclopropane fatty acid content is a major factor in acid resistance of Escherichia coli. Mol. Microbiol..

[18] Jiang X., Duan Y., Zhou B., Guo Q., Wang H., Hang X. (2019). The cyclopropane fatty acid synthase mediates antibiotic resistance and gastric colonization of Helicobacter pylori. J. Bacteriol..

[19] Lolli V., Marseglia A., Palla G., Zanardi E., Caligiani A. (2018). Determination of cyclopropane fatty acids in food of animal origin by 1H NMR. J. Anal. Methods Chem..

[20] Mika A., Stepnowski P., Chmielewski M., Malgorzewicz S., Kaska L., Proczko M. (2016). Increased serum level of cyclopropaneoctanoic acid 2-Hexyl in patients with hypertriglyceridemia-related disorders. Lipids.

[21] Paton C.M., Vaughan R.A., Selen Alpergin E.S., Assadi-Porter F., Dowd M.K. (2017). Dihydrosterculic acid from cottonseed oil suppresses desaturase activity and improves liver metabolomic profiles of high-fat–fed mice. Nutr. Res..

[22] Lolli V., Dall’Asta M., Rio D.D., Palla G., Caligiani A. (2019). Presence of cyclopropane fatty acids in foods and estimation of dietary intake in the Italian population. Int. J. Food Sci. Nutr..

[23] Colosimo D.A., Kohn J.A., Luo P.M., Piscotta F.J., Han S.M., Pickard A.J. (2019). Mapping interactions of microbial metabolites with human G-Protein-Coupled receptors. Cell Host Microbe.

[24] Sobhi H.F., Mercer K.E., Lan R.S., Yeruva L., Have G.A.M.T., Deutz N.E.P. (2024). Novel odd-chain cyclopropane fatty acids: detection in a mammalian lipidome and uptake by hepatosplanchnic tissues. J. Lipid Res..

[25] Sledzinski T., Mika A., Stepnowski P., Proczko-Markuszewska M., Kaska L., Stefaniak T. (2013). Identification of cyclopropaneoctanoic acid 2-Hexyl in human adipose tissue and serum. Lipids.

[26] Lolli V., Dall’Asta M., Caligiani A., Del Rio D., de la Fuente M.A., Gómez-Cortés P. (2022). Detection of cyclopropane fatty acids in human breastmilk by GC-MS. J. Food Compost. Anal..

[27] Lolli V., Dall’Asta M., Del Rio D., Caligiani A. (2020). Identification of cyclopropane fatty acids in human plasma after controlled dietary intake of specific foods. Nutrients.

[28] Sobhi H.F., Zhao X., Plomgaard P., Hoene M., Hansen J.S., Karus B. (2020). Identification and regulation of the xenometabolite derivatives cis- and trans-3,4-methylene-heptanoylcarnitine in plasma and skeletal muscle of exercising humans. Am. J. Physiol. Endocrinol. Metab..

[29] Jaladanki C.K., He Y., Zhao L.N., Maurer-Stroh S., Loo L.-H., Song H. (2021). Virtual screening of potentially endocrine-disrupting chemicals against nuclear receptors and its application to identify PPARγ-bound fatty acids. Arch. Toxicol..

[30] Li J., Abel R., Zhu K., Cao Y., Zhao S., Friesner R.A. (2011). The VSGB 2.0 model: a next generation energy model for high resolution protein structure modeling. Proteins.

[31] La Paglia L., Listì A., Caruso S., Amodeo V., Passiglia F., Bazan V. (2017). Potential role of ANGPTL4 in the cross talk between metabolism and cancer through PPAR signaling pathway. PPAR Res..

[32] Seelig A., Seelig J. (1977). Effect of a single cis double bond on the structure of a phospholipid bilayer. Biochemistry.

[33] Poger D., Mark A.E. (2015). A ring to rule them all: the effect of cyclopropane fatty acids on the fluidity of lipid bilayers. J. Phys. Chem. B.

[34] Róg T., Murzyn K., Gurbiel R., Takaoka Y., Kusumi A., Pasenkiewicz-Gierula M. (2004). Effects of phospholipid unsaturation on the bilayer nonpolar region. J. Lipid Res..

[35] Loffhagen N., Härtig C., Geyer W., Voyevoda M., Harms H. (2007). Competition between cis, trans and cyclopropane fatty acid formation and its impact on membrane fluidity. Eng. Life Sci..

[36] Hagve T.-A. (1988). Effects of unsaturated fatty acids on cell membrane functions. Scand. J. Clin. Lab. Invest..

[37] Silvius J.R., McElhaney R.N. (1979). Effects of phospholipid acylchain structure on thermotropic phase properties. 2: phosphatidylcholines with unsaturated or cyclopropane acyl chains. Chem. Phys. Lipids.

[38] Grogan D.W., Cronan J.E. (1997). Cyclopropane ring formation in membrane lipids of bacteria. Microbiol. Mol. Biol. Rev..

[39] Desvergne B., Wahli W. (1999). Peroxisome proliferator-activated receptors: Nuclear Control of Metabolism. Endocr. Rev..

[40] Banner C., Göttlicher M., Widmark E., Sjövall J., Rafter J., Gustafsson J. (1993). A systematic analytical chemistry/cell assay approach to isolate activators of orphan nuclear receptors from biological extracts: characterization of peroxisome proliferator-activated receptor activators in plasma. J. Lipid Res..

[41] Xu H.E., Lambert M.H., Montana V.G., Parks D.J., Blanchard S.G., Brown P.J. (1999). Molecular recognition of fatty acids by peroxisome proliferator–activated receptors. Mol. Cell.

[42] Kliewer S.A., Sundseth S.S., Jones S.A., Brown P.J., Wisely G.B., Koble C.S. (1997). Fatty acids and eicosanoids regulate gene expression through direct interactions with peroxisome proliferator-activated receptors α and γ. Proc. Natl. Acad. Sci. U. S. A..

[43] Xu H.E., Lambert M.H., Montana V.G., Plunket K.D., Moore L.B., Collins J.L. (2001). Structural determinants of ligand binding selectivity between the peroxisome proliferator-activated receptors. Proc. Natl. Acad. Sci. U. S. A..

[44] Bougarne N., Weyers B., Desmet S.J., Deckers J., Ray D.W., Staels B. (2018). Molecular actions of PPARα in lipid metabolism and inflammation. Endocr. Rev..

[45] Itoh T., Fairall L., Amin K., Inaba Y., Szanto A., Balint B.L. (2008). Structural basis for the activation of PPARγ by oxidized fatty acids. Nat. Struct. Mol. Biol..

[46] Nolte R.T., Wisely G.B., Westin S., Cobb J.E., Lambert M.H., Kurokawa R. (1998). Ligand binding and co-activator assembly of the peroxisome proliferator-activated receptor-g. Nature.

[47] Feige J.N., Gelman L., Michalik L., Desvergne B., Wahli W. (2006). From molecular action to physiological outputs: peroxisome proliferator-activated receptors are nuclear receptors at the crossroads of key cellular functions. Prog. Lipid Res..

[48] Kuwabara N., Oyama T., Tomioka D., Ohashi M., Yanagisawa J., Shimizu T. (2012). Peroxisome proliferator-activated receptors (PPARs) have multiple binding points that accommodate ligands in various conformations: phenylpropanoic acid-type PPAR ligands bind to PPAR in different conformations, depending on the subtype. J. Med. Chem..

[49] Bruning J.B., Chalmers M.J., Prasad S., Busby S.A., Kamenecka T.M., He Y. (2007). Partial agonists activate PPARγ using a helix 12 independent mechanism. Structure.

[50] Yoon J.C., Chickering T.W., Rosen E.D., Dussault B., Qin Y., Soukas A. (2000). Peroxisome proliferator-activated receptor " target gene encoding A novel angiopoietin-related protein associated with adipose differentiation. Mol. Cell Biol..

[51] Kaddatz K., Adhikary T., Finkernagel F., Meissner W., MÜller-BrÜsselbach S., MÜller R. (2010). Transcriptional profiling identifies functional interactions of TGFβ and PPARβ/δ signaling: synergistic induction of ANGPTL4 transcription[S]. J. Biol. Chem..

[52] Kersten S., Mandard S., Tan N.S., Escher P., Metzger D., Chambon P. (2000). Characterization of the fasting-induced adipose factor FIAF, a novel peroxisome proliferator-activated receptor target gene. J. Biol. Chem..

[53] Georgiadi A., Lichtenstein L., Degenhardt T., Boekschoten M.V., van Bilsen M., Desvergne B. (2010). Induction of cardiac Angptl4 by dietary fatty acids is mediated by peroxisome proliferator-activated receptor β/δ and protects against fatty acid–induced oxidative stress. Circ. Res..

[54] Staiger H., Haas C., Machann J., Werner R., Weisser M., Schick F. (2009). Muscle-derived angiopoietin-like protein 4 is induced by fatty acids via peroxisome proliferator–activated receptor (PPAR)-δ and is of metabolic relevance in humans. Diabetes.

[55] Abdelmagid S.A., Clarke S.E., Nielsen D.E., Badawi A., El-Sohemy A., Mutch D.M. (2015). Comprehensive profiling of plasma fatty acid concentrations in young healthy Canadian adults. PLoS One.

[56] Pap A., Cuaranta-Monroy I., Peloquin M., Nagy L. (2016). Is the mouse a good model of human PPARγ-Related metabolic diseases?. Int. J. Mol. Sci..

[57] Rigamonti E., Chinetti-Gbaguidi G., Staels B. (2008). Regulation of macrophage functions by PPAR-alpha, PPAR-gamma, and LXRs in mice and men. Arterioscler. Thromb. Vasc. Biol..

[58] Atalay Ekiner S., Gęgotek A., Skrzydlewska E. (2024). Inflammasome activity regulation by PUFA metabolites. Front. Immunol..

[59] Kamata S, Oyama T, Saito K, Honda A, Yamamoto Y, Suda K (2020). PPARα ligand-binding domain structures with endogenous fatty acids and fibrates. iScience.

[60] Jumper J, Evans R, Pritzel A, Green T, Figurnov M, Ronneberger O (2021). Highly accurate protein structure prediction with AlphaFold. Nature.

[61] Wu CC, Baiga TJ, Downes M, La Clair JJ, Atkins AR, Richard SB (2017). Structural basis for specific ligation of the peroxisome proliferator-activated receptor δ. Proc. Natl. Acad. Sci..

[62] Fyffe SA, Alphey MS, Buetow L, Smith TK, Ferguson MAJ, Sørensen MD (2006). Recombinant human PPAR-β/δ ligand-binding domain is locked in an activated conformation by endogenous fatty acids. J. Mol. Biol..

[63] Willems S, Gellrich L, Chaikuad A, Kluge S, Werz O, Heering J (2021). Endogenous vitamin E metabolites mediate allosteric PPARγ activation with unprecedented co-regulatory interactions. Cell Chem. Biol..

[64] Shang J, Brust R, Mosure SA, Bass J, Munoz-Tello P, Lin H (2018). Cooperative cobinding of synthetic and natural ligands to the nuclear receptor PPARγ. eLife.

[65] Useini A, Schwerin IK, Künze G, Sträter N (2024). Structural studies on the binding mode of bisphenols to PPARγ. Biomolecules.

