# Supplementary material for: Cyclopropane xenolipids resemble monounsaturated fatty acids and modulate peroxisome proliferator-activated receptors
Source: J Lipid Res. 2025 Sep 5;66(11):100896. doi: 10.1016/j.jlr.2025.100896 (PMC12605127; doi:10.1016/j.jlr.2025.100896)
Supplement: Supplementary Materials [file mmc1.docx]

# SUPPLEMENTARY FIGURES

Supplementary Figure 1. Number of distinct conformers for each cyclopropane fatty acid (CpFA), monounsaturated fatty acid (MUFA), and saturated fatty acid (SFA)

#### Each dot represents a specific fatty acid, with its Lipid ID on the x-axis corresponding to the Lipid ID in Supplementary Table 2. A total of 429 fatty acids are included. Vertical lines separate fatty acids by their carbon chain length. C, carbon; CpFA, cyclopropane fatty acids; MUFA, mono-unsaturated fatty acid; SFA, saturated fatty acid

Supplementary Figure 2. Bendability and conformational diversity of all CpFAs, MUFAs, and SFAs

#### (A) End-to-end distances and (B) radius of gyration (both in Å) and (C) convex hull area (measuring the area covered by the lipid in a PCA space in Å^2^) according to the length of their carbon backbone and the position of the double bond for MUFAs (in blue) and the cyclopropane ring for CpFAs (in orange). Data are expressed as mean ± standard deviation (shaded areas) for A and B, and area ± standard error of the mean for (C). Two-way ANOVA and Tukey’s HSD post-hoc tests were used to compare values across lipid types (CpFA, MUFA, SFA) and position of double bond/unsaturation (2–17) for each lipid length independently; compact letters denote significant differences (q < 0.05) between groups, with shared "a" for CpFA vs. MUFA, "b" for CpFA vs. SFA, and "c" for MUFA vs. SFA. The mean and standard deviation values for saturated lipids (in black) were projected across the plot as references and used for statistical comparisons. CpFA, cyclopropane fatty acids; MUFA, mono-unsaturated fatty acid; PCA, principal component analysis; SFA, saturated fatty acid

Supplementary Figure 3. Alignment of human PPAR structures and impact on free binding energies for 429 known and theoretical SFAs, CpFAs, and MUFAs

#### (A-C) Alignment of three-dimensional structures of PPARα, PPARδ, and PPARγ, respectively. Color coding represents different structures (e.g., 6KB3, 6LX8 for PPARα), shown in stick representation. Distances between structures shown in tables are expressed in Å. (D-F) Principal component analysis (PCA) plots illustrating the variance in the calculated free binding energies of each lipid toward individual structures within PPAR isoforms. Each point represents the binding energy for a given FA with a specific PPAR isoform structure; 429 lipids were analyzed. Ellipses indicate 95% confidence intervals. CpFA, cyclopropane fatty acids; MUFA, mono-unsaturated fatty acid; PCA, principal component analysis; SFA, saturated fatty acid

Supplementary Figure 4. Individual binding energy values of 429 SFAs, CpFAs, and MUFAs toward human PPARα, PPARδ, and PPARγ

#### MM/GBSA free binding energies (in kcal·mol^-1^) for all 429 lipids across all three PPAR subunits. The top 5% of lipids with the strongest predicted binding (lowest free binding energies) are highlighted in bold with white borders, while the values for lipids selected for follow-up in vitro experiments are displayed with white text. CpFA, cyclopropane fatty acids; MUFA, mono-unsaturated fatty acid; PPAR: peroxisome proliferator-activated receptors; SFA, saturated fatty acid

Supplementary Figure 5. PCA visualization of free binding energies to human PPAR isoforms – impact of lipid structural characteristics and energy components

#### PCA representation of individual binding energy values of 429 SFAs, CpFAs, and MUFAs, with overlayed variables that contributed to the overall projections. Black squares represent SFAs, blue circles MUFAs, and orange triangles CpFAs. CpFA, cyclopropane fatty acids; MUFA, mono-unsaturated fatty acid; PCA, principal component analysis; PPAR: peroxisome proliferator-activated receptors; SFA, saturated fatty acid

Supplementary Figure 6. PPARγ binding results using TR-FRET binding assays.

#### CpFA, cyclopropane fatty acids; EC_50_: half maximal effective concentration; Max: maximum fitted intensity; MUFA, mono-unsaturated fatty acid; PCA, principal component analysis; PPAR: peroxisome proliferator-activated receptors; SFA, saturated fatty acid

Supplementary Figure 7. 490 nm and 520 nm responses for the PPARα TR-FRET binding assay.

#### CpFA, cyclopropane fatty acids; MUFA, mono-unsaturated fatty acid; PPAR: peroxisome proliferator-activated receptors; SFA, saturated fatty acid

Supplementary Figure 8. 490 nm and 520 nm responses for the PPARδ TR-FRET binding assay.

#### CpFA, cyclopropane fatty acids; MUFA, mono-unsaturated fatty acid; PPAR: peroxisome proliferator-activated receptors; SFA, saturated fatty acid

Supplementary Figure 9. 490 nm and 520 nm responses for the PPARγ TR-FRET binding assay.

#### CpFA, cyclopropane fatty acids; MUFA, mono-unsaturated fatty acid; PPAR: peroxisome proliferator-activated receptors; SFA, saturated fatty acid.

Supplementary Figure 10. *In vitro* binding results mostly corroborate predicted binding affinities obtained *in silico*.

#### (A-B) Pearson’s correlations between in vitro E_max_ values and in silico binding energies for PPARα and PPARδ, respectively. (C-D) Pearson’s and Spearman’s correlations between in vitro EC_50_ values and in silico binding energies for PPARα and PPARδ, respectively.

Supplementary Figure 11. Distribution of lipid categories within conformational clusters per PPAR isoform

#### (A–C) Two-dimensional coordinates of head-group positions for all conformers generated from the 14 lipids tested in vitro (see Supplementary Table 3), shown separately for CpFAs (left), MUFAs (middle), and SFAs (right) within the ligand-binding domains of PPARα (A), PPARδ (B), and PPARγ (C). Conformers are grouped by spatial proximity using k-means clustering. Density plots above each panel depict the relative distribution of conformers from each cluster within a given lipid category. Lipid category enrichment across clusters was evaluated using Pearson’s chi-squared test, and the degree of separation between clusters was assessed via MANOVA. This visualization highlights receptor-specific clustering patterns associated with each lipid class.

Supplementary Figure 12. Residue interaction fingerprint across fatty acid category.

#### (A-C) Heatmap showing the average number of lipid-residue interaction(s) for all 14 lipids tested in vitro (see Supplementary Table 3), categorized per lipid class (CpFA, MUFA and SFA), for each ligand-binding domain of PPARα (A), PPARδ (B), and PPARγ (C). The total interaction count per residue represents the sum of nine interaction types: Contact, Backbone, Sidechain, Polar, Hydrophobic, Acceptor, Donor, Aromatic, and Charged. Interaction values were binned into intervals for visual clarity. White indicates the absence of detectable interactions.

Supplementary Figure 13. Residue interaction fingerprint across clusters of lipid positions within the receptors’ LBDs.

#### (A-C) Heatmap showing the average number of lipid-residue interaction(s) for all 14 lipids tested in vitro (see Supplementary Table 3), categorized per clusters of lipid positions, for each ligand-binding domain of PPARα (A), PPARδ (B), and PPARγ (C). The total interaction count per residue represents the sum of nine interaction types: Contact, Backbone, Sidechain, Polar, Hydrophobic, Acceptor, Donor, Aromatic, and Charged. Interaction values were binned into intervals for visual clarity. White indicates the absence of detectable interactions.

Supplementary Figure 14. Differential residue interaction patterns across structural clusters for each PPAR isoform

#### Bar plots displaying the signed differences in average number of lipid-residue interaction scores between selected cluster pairs for PPARα, PPARδ, and PPARγ, based on all 14 lipids tested *in vitro*. Each bar represents a residue and corresponds to the difference in mean interaction score between two clusters (positive = stronger in the second cluster; negative = stronger in the first). Interaction scores represent the sum of nine interaction types: Contact, Backbone, Sidechain, Polar, Hydrophobic, Acceptor, Donor, Aromatic, and Charged. Panels (A-B) show the comparison between cluster #1 and #2 for PPARα and PPARδ, respectively. Panel C display all six pairwise comparisons between clusters #1-#4 for PPARγ. Bars are colored by statistical significance after FDR correction (dark grey: q < 0.05; light grey: not significant). Only the top 20 residues with the largest absolute differences are shown for each comparison.

Supplementary Figure 15. Dose-dependent induction of *Angptl-4* expression by PPAR agonists in 3T3-L1 preadipocyte cells

#### Bar-plots showing *Angptl-4* mRNA fold-change following treatment with increasing concentrations (0.1, 1, and 10 µM) of selective agonists for PPARα (GW7647), PPARδ (GW501516), and PPARγ (Rosiglitazone). Gene expression was measured by RT-qPCR and normalized to vehicle-treated controls. Statistical significance was assessed using Kruskal–Wallis rank sum tests, followed by Dunn’s post hoc tests with Holm correction versus vehicle. Asterisks indicate significance levels: p < 0.05 (*), < 0.0001 (***); ns = not significant. Error bars represent standard error of the mean (SEM). Angptl-4, Angiopoietin-like protein 4; PPAR, peroxisome proliferator-activated receptors.

Supplementary Figure 16. Repression of Angptl-4 expression by PPAR antagonists in 3T3-L1 preadipocyte cells

#### Bar-plots showing *Angptl-4* mRNA fold-change in response to PPAR agonists alone or in combination with their respective antagonists for PPARα (GW7647 with GW6471), PPARδ (GW501516 with GSK3787), and PPARγ (Rosiglitazone with T0070907). Both agonists and antagonists were used at 10 µM. Conditions with a light grey background are normalized to their respective vehicle controls, while those with navy blue backgrounds represent recalculated values normalized against the antagonist-only condition. Error bars represent standard error of the mean (SEM). Angptl-4, Angiopoietin-like protein 4; PPAR, peroxisome proliferator-activated receptors.

# SUPPLEMENTARY TABLES

Supplementary Table 1. Characteristics of the human PPARs 3D structures used for the *in silico* study

*: this chain was selected for the docking work. CID: compound identifier; LBD: ligand binding domain; PDB: Protein Data Bank; PPAR: Peroxisome proliferator-activated receptor

| **PDB** | **PPAR** | **Species** | **Portion** | **Chain(s)** | **Length** | **Res (Å)** | **Date released** | **Ligand** | **Ref** |
| --- | --- | --- | --- | --- | --- | --- | --- | --- | --- |
| 6KB3 | Alpha | *Homo sapiens* | LBD | A | 273 | 1.45 | 11/2020 | GW7647 | (1) |
| 6LX8 | Alpha |  | LBD | A | 273 | 1.54 | 11/2020 | Oleic acid | (1) |
| 6LXA | Alpha |  | LBD | A | 273 | 1.23 | 11/2020 | Eicosapentaenoic acid | (1) |
| 7BQ0 | Alpha |  | LBD | A | 273 | 1.77 | 11/2020 | Fenofibrate | (1) |
| Q07869 | Alpha |  | Full | N/A | 468 | N/A | 11/2022 | None | (2) |
| 5U3Q | Delta | *Homo sapiens* | LBD | A*, B | 272 | 1.50 | 03/2017 | CID 117630078 | (3) |
| 5Y7X | Delta |  | LBD | A*, B | 292 | 1.70 | 08/2018 | CID 11620299 | - |
| 2AWH | Delta |  | LBD | A | 268 | 2 | 02/2006 | cis-Vaccenic acid | (4) |
| 5U3S | Delta |  | LBD | A*, B | 272 | 2 | 03/2017 | CID 86280885 | (3) |
| Q03181 | Delta |  | Full | N/A | 441 | N/A | 11/2022 | None | (2) |
| 7AWC | Gamma | *Homo sapiens* | LBD | A | 277 | 1.74 | 11/2020 | Rosiglitazone | (5) |
| 6D8X | Gamma |  | LBD | A | 297 | 1.90 | 05/2019 | GW1929 | - |
| 6MD0 | Gamma |  | LBD | A | 275 | 1.95 | 01/2019 | Oleic acid | (6) |
| 9F7W | Gamma |  | LBD | A | 283 | 1.25 | 07/2024 | Bisphenol A | (7) |
| P37231 | Gamma |  | Full | N/A | 505 | N/A | 11/2022 | None | (2) |

Supplementary Table 2. Characteristics of all 429 putative lipid structures used for the *in silico* study

CID: compound identifier; CpFA: cyclopropane fatty acid; IUPAC: international union of pure and applied chemistry name; MUFA: mono-unsaturated fatty acid; SFA: saturated fatty acid; SMILE: simplified molecular input line entry system.

**EXTERNAL FILE**

Supplementary Table 3. List of lipids used for the *in vitro* studies

Lipid ID corresponds to the Lipid.ID column in Supplementary Table 2. CAS: chemical abstracts service; CpFA: cyclopropane fatty acid; MUFA: mono-unsaturated fatty acid; SFA: saturated fatty acid.

| **Lipid ID** | **Cat** | **Name** | | **aka** | **CAS n°** | **Provider** | **Ref #** | **Purity** |
| --- | --- | --- | --- | --- | --- | --- | --- | --- |
| 306 | CpFA | C18∆1 *cis*-11 | (11S,12R)-11,12-methyleneoctadecanoic acid | Lactobacillic acid | 503-06-0 | Cayman | 10012556 | >98% |
| 98 | MUFA | C18:1 *cis*-11 | (11Z)-octadec-11-enoic acid | Vaccenic acid | 506-17-2 | Cayman | 20023 | >98% |
| 304 | CpFA | C18∆1 *cis*-9 | (9S,10R)-9,10-methyleneoctadecanoic acid | Dihydrosterculic acid | 4675-61-0 | Cayman | 24824 | >98% |
| 96 | MUFA | C18:1 *cis*-9 | (9Z)-octadec-9-enoic acid | Oleic acid | 112-80-1 | Cayman | 90260 | >98% |
| 7 | SFA | C18 | Octadecanoic acid | Stearic acid | 57-11-4 | Cayman | 10011298 | >98% |
| 293 | CpFA | C17∆1 *cis*-13 | (13S,14R)-13,14-methyleneheptadecanoic acid | - | - | Larodan AB | Custom | >98% |
| 85 | MUFA | C17:1 *cis*-13 | (13Z)-heptadec-13-enoic acid | Thudichumic acid | - | Larodan AB | Custom | >99% |
| 6 | SFA | C17 | Heptadecanoic acid | Margaric acid | 506-12-7 | Cayman | 19722 | >95% |
| 275 | CpFA | C16∆1 *cis*-9 | (9S,10R)-9,10-methylenehexadecanoic acid | - | 5618-00-8 | Larodan AB | Custom | >99% |
| 67 | MUFA | C16:1 *cis*-9 | (9Z)-hexadec-9-enoic acid | Palmitoleic acid | 373-49-9 | Cayman | 10009871 | >99% |
| 5 | SFA | C16 | Hexadecanoic acid | Palmitic acid | 57-10-3 | Cayman | 10006627 | >98% |
| 264 | CpFA | C15∆1 *cis*-11 | (11S,12R)-11,12-methylenepentadecanoic acid | - | - | Larodan AB | Custom | >99% |
| 56 | MUFA | C15:1 *cis*-11 | (11Z)-pentadec-11-enoic acid | Liebigeic acid | - | Larodan AB | Custom | >99% |
| 4 | SFA | C15 | Pentadecanoic acid | Pentadecylic acid | 1002-84-2 | Cayman | 17399 | >98% |

Supplementary Table 4. List PPAR agonists and antagonists used for the *in vitro* studies

CAS: chemical abstracts service; CpFA: cyclopropane fatty acid; MUFA: mono-unsaturated fatty acid; PPAR: peroxisome proliferator activated receptor; SFA: saturated fatty acid.

| **Category** | **Name** | **CAS n°** | **Provider** | **Ref #** | **Purity** |
| --- | --- | --- | --- | --- | --- |
| PPARα Agonist | GW7647 | 265129-71-3 | Cayman | 10008613 | >98% |
| PPARα Antagonist | GW6471 | 880635-03-0 | Cayman | 11697 | >98% |
| PPARδ Agonist | GW501516 | 317318-70-0 | Cayman | 10004272 | >98% |
| PPARδ Antagonist | GSK3787 | 188591-46-0 | Cayman | 15219 | >98% |
| PPARγ Agonist | Rosiglitazone | 122320-73-4 | Cayman | 71740 | >98% |
| PPARγ Antagonist | T0070907 | 313516-66-4 | Cayman | 10026 | >98% |

Supplementary Table 5. List of primers used for the in vitro gene expression assay in 3T3-L1 preadipocytes

| **Target gene** | **Sequence** | **Direction** | **Amplicon size** | **Species** |
| --- | --- | --- | --- | --- |
| Angptl4 | CATCCTGGGACGAGATGAACT | F | 136 bp | *Mus musculus* |
|  | TGACAAGCGTTACCACAGGC | R |  |  |
| Rn18S | GGTGCATGGCCGTTCTTA | F | 70 bp | *Mus musculus* |
|  | TGCCAGAGTCTCGTTCGTTA | R |  |  |
| Rplp0 | TGAGATTCGGGATATGCTGTTGG | F | 204 bp | *Mus musculus* |
|  | CGGGTCCTAGACCAGTGTTCT | R |  |  |

# Supplementary References

1. Kamata S, Oyama T, Saito K, Honda A, Yamamoto Y, Suda K, et al. PPARα Ligand-Binding Domain Structures with Endogenous Fatty Acids and Fibrates. iScience. 2020 Nov 20;23(11):101727.

2. Jumper J, Evans R, Pritzel A, Green T, Figurnov M, Ronneberger O, et al. Highly accurate protein structure prediction with AlphaFold. Nature. 2021 Aug;596(7873):583–9.

3. Wu CC, Baiga TJ, Downes M, La Clair JJ, Atkins AR, Richard SB, et al. Structural basis for specific ligation of the peroxisome proliferator-activated receptor δ. Proc Natl Acad Sci. 2017 Mar 28;114(13):E2563–70.

4. Fyffe SA, Alphey MS, Buetow L, Smith TK, Ferguson MAJ, Sørensen MD, et al. Recombinant Human PPAR-β/δ Ligand-binding Domain is Locked in an Activated Conformation by Endogenous Fatty Acids. J Mol Biol. 2006 Mar 3;356(4):1005–13.

5. Willems S, Gellrich L, Chaikuad A, Kluge S, Werz O, Heering J, et al. Endogenous vitamin E metabolites mediate allosteric PPARγ activation with unprecedented co-regulatory interactions. Cell Chem Biol. 2021 Oct 21;28(10):1489-1500.e8.

6. Shang J, Brust R, Mosure SA, Bass J, Munoz-Tello P, Lin H, et al. Cooperative cobinding of synthetic and natural ligands to the nuclear receptor PPARγ. eLife. 2018;7.

7. Useini A, Schwerin IK, Künze G, Sträter N. Structural Studies on the Binding Mode of Bisphenols to PPARγ. Biomolecules. 2024 Jun;14(6):640.
