# Supplementary material for: Cyclopropane xenolipids resemble monounsaturated fatty acids and modulate peroxisome proliferator-activated receptors
Source: J Lipid Res. 2025 Sep 5;66(11):100896. doi: 10.1016/j.jlr.2025.100896 (PMC12605127; doi:10.1016/j.jlr.2025.100896)
Supplement: Supplementary Figures [file mmc3.pdf]

Cyclopropane xenolipids resemble monounsaturated fatty acids and modulate peroxisome proliferator-activated receptors

Jean Debédat, Lorena Pastor, Trina A. Knotts, Jordan G. Pitman, Kristine Griffett, Sean H. Adams

Supplementary figures

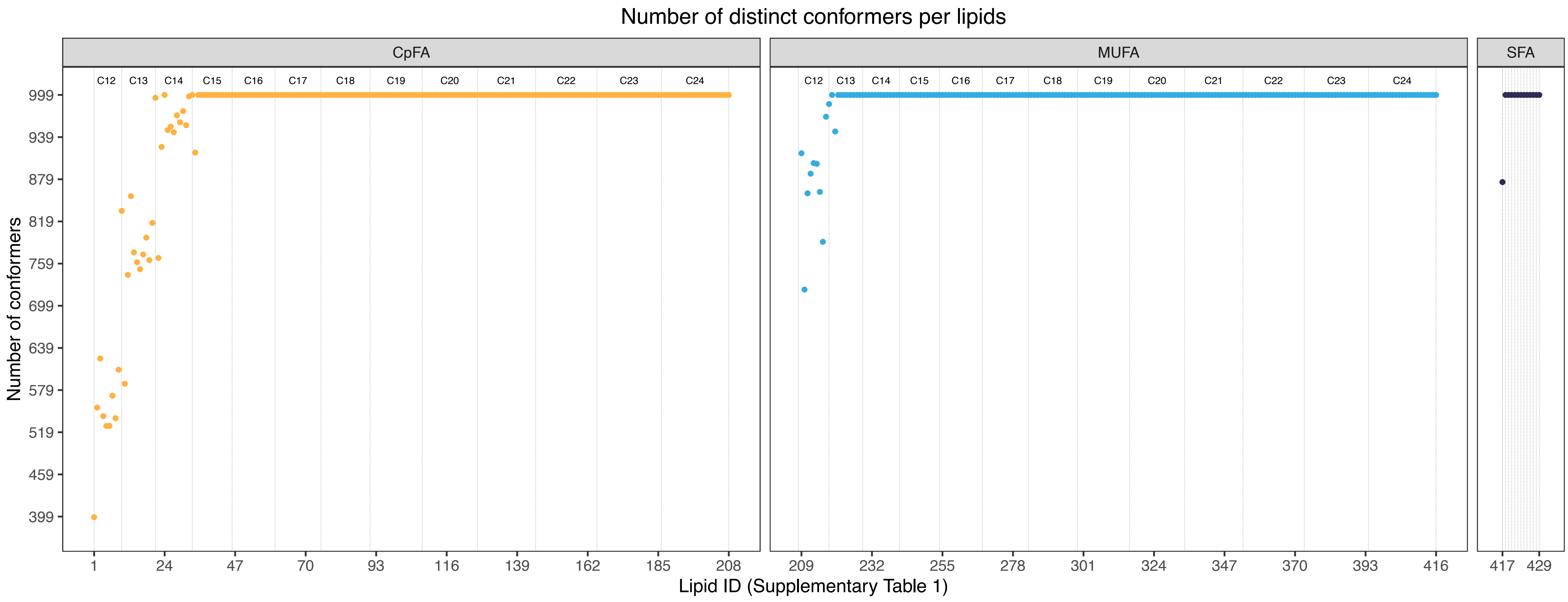

Supplementary Figure 1. Number of distinct conformers for each cyclopropane fatty acid (CpFA), monounsaturated fatty acid (MUFA), and saturated fatty acid (SFA)

Each dot represents a specific fatty acid, with its Lipid ID on the x-axis corresponding to the Lipid ID in Supplementary Table 2. A total of 429 fatty acids are included. Vertical lines separate fatty acids by their carbon chain length. C, carbon; CpFA, cyclopropane fatty acids; MUFA, mono-unsaturated fatty acid; SFA, saturated fatty acid

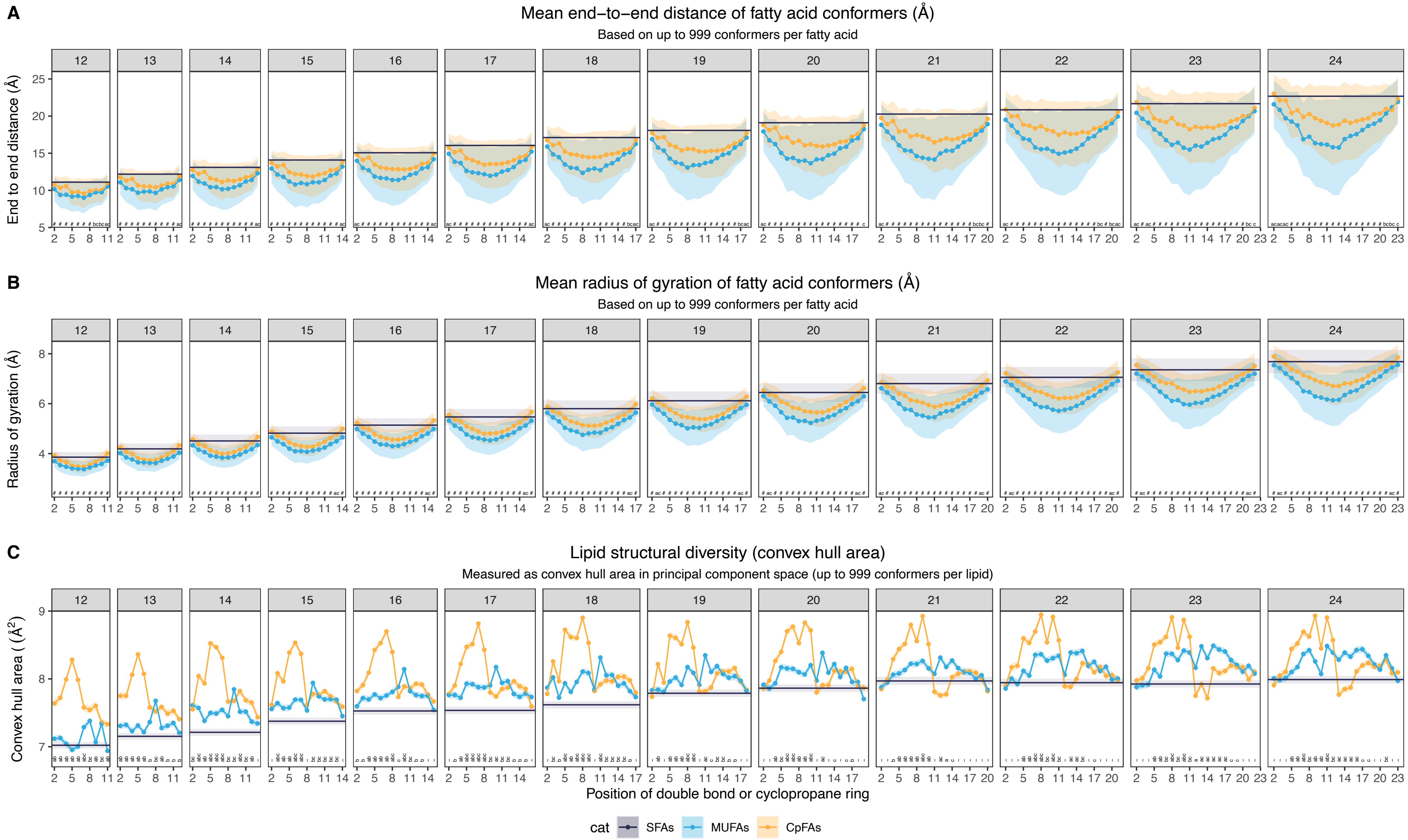

Supplementary Figure 2. Bendability and conformational diversity of all CpFAs, MUFAs, and SFAs

(A) End-to-end distances and (B) radius of gyration (both in Å) and (C) convex hull area (measuring the area covered by the lipid in a PCA space in Å2) according to the length of their carbon backbone and the position of the double bond for MUFAs (in blue) and the cyclopropane ring for CpFAs (in orange). Data are expressed as mean ± standard deviation (shaded areas) for A and B, and area ± standard error of the mean for (C). Two-way ANOVA and Tukey's HSD post-hoc tests were used to compare values across lipid types (CpFA, MUFA, SFA) and position of double bond/unsaturation (2–17) for each lipid length independently; compact letters denote significant differences ($q < 0.05$) between groups, with shared "a" for CpFA vs. MUFA, "b" for CpFA vs. SFA, and "c" for MUFA vs. SFA. The mean and standard deviation values for saturated lipids (in black) were projected across the plot as references and used for statistical comparisons. CpFA, cyclopropane fatty acids; MUFA, mono-unsaturated fatty acid; PCA, principal component analysis; SFA, saturated fatty acid

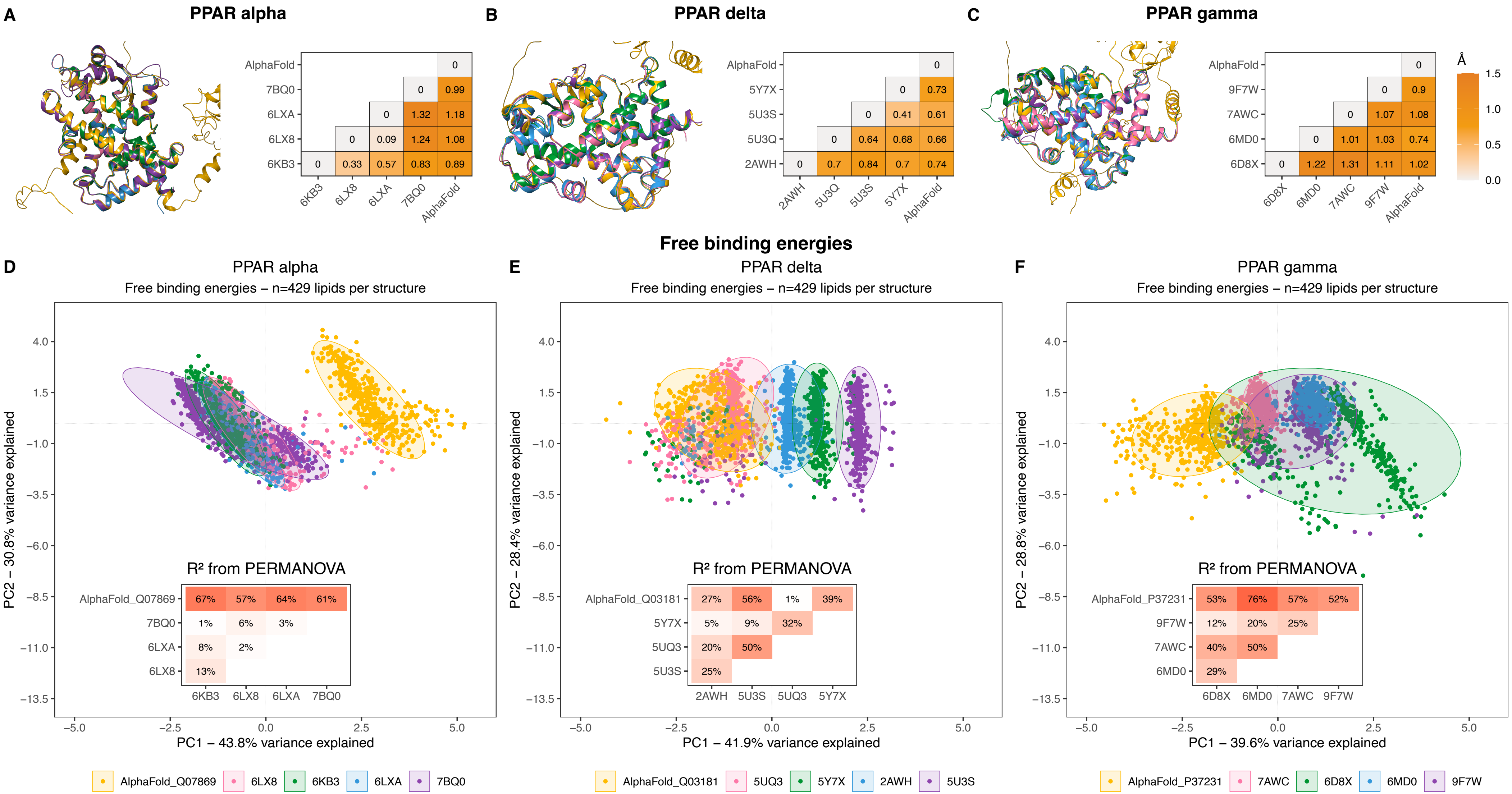

Supplementary Figure 3. Alignment of human PPAR structures and impact on free binding energies for 429 known and theoretical SFAs, CpFAs, and MUFAs

(A-C) Alignment of three-dimensional structures of PPARα, PPARδ, and PPARγ, respectively. Color coding represents different structures (e.g., 6KB3, 6LX8 for PPARα), shown in stick representation. Distances between structures shown in tables are expressed in Å. (D-F) Principal component analysis (PCA) plots illustrating the variance in the calculated free binding energies of each lipid toward individual structures within PPAR isoforms. Each point represents the binding energy for a given FA with a specific PPAR isoform structure; 429 lipids were analyzed. Ellipses indicate 95% confidence intervals. CpFA, cyclopropane fatty acids; MUFA, mono-unsaturated fatty acid; PCA, principal component analysis; SFA, saturated fatty acid

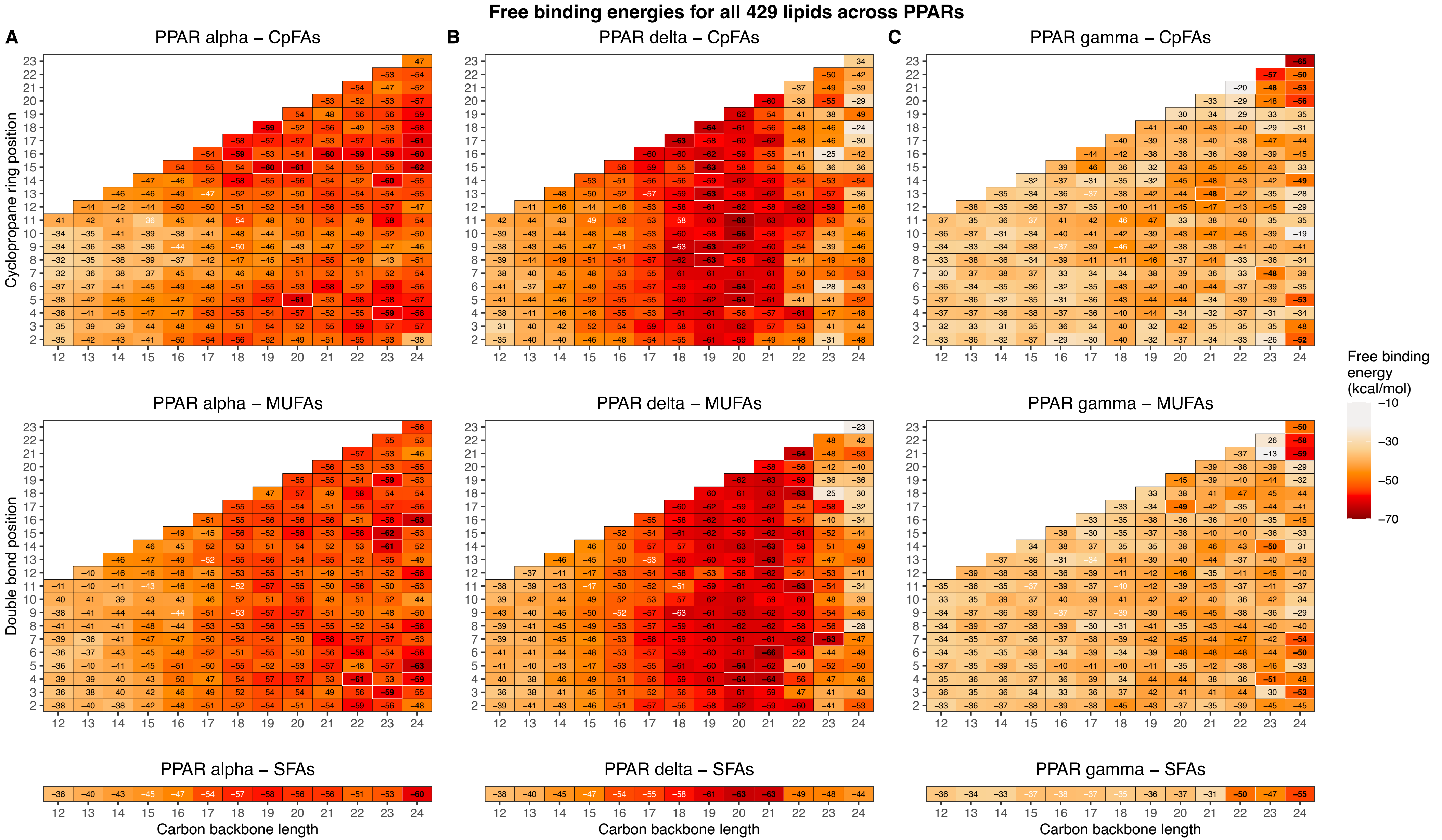

Supplementary Figure 4. Individual binding energy values of 429 SFAs, CpFAs, and MUFAs toward human PPARα, PPARδ, and PPARγ

MM/GBSA free binding energies (in kcal·mol-1) for all 429 lipids across all three PPAR subunits. The top 5% of lipids with the strongest predicted binding (lowest free binding energies) are highlighted in bold with white borders, while the values for lipids selected for follow-up in vitro experiments are displayed with white text. CpFA, cyclopropane fatty acids; MUFA, mono-unsaturated fatty acid; PPAR: peroxisome proliferator-activated receptors; SFA, saturated fatty acid

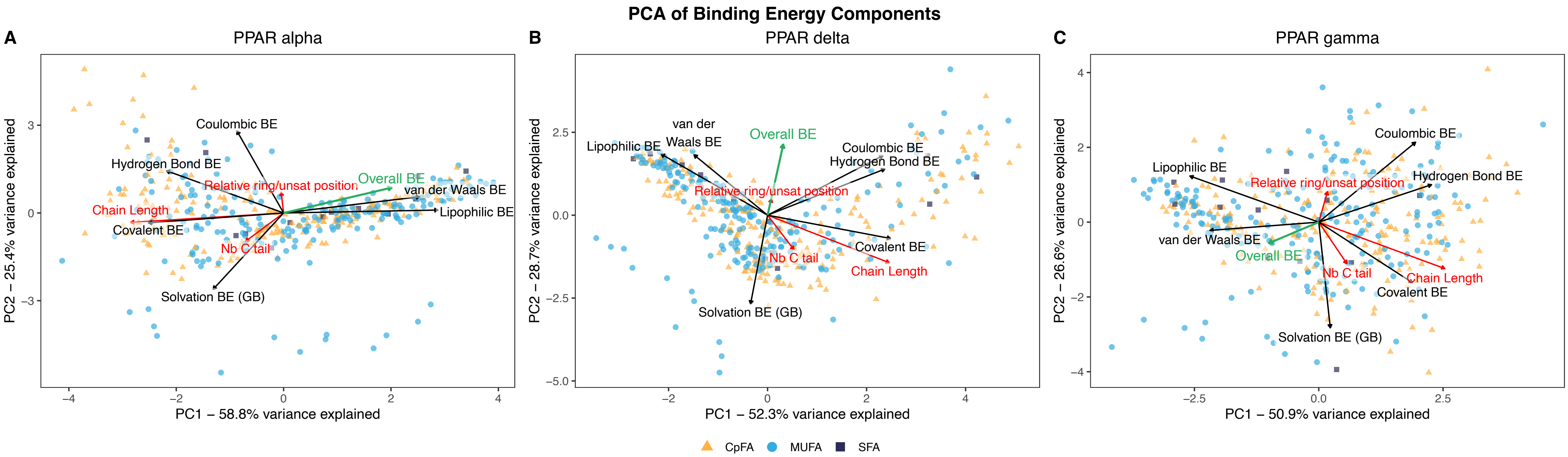

Supplementary Figure 5. PCA visualization of free binding energies to human PPAR isoforms – impact of lipid structural characteristics and energy components

PCA representation of individual binding energy values of 429 SFAs, CpFAs, and MUFAs, with overlayed variables that contributed to the overall projections. Black squares represent SFAs, blue circles MUFAs, and orange triangles CpFAs. CpFA, cyclopropane fatty acids; MUFA, mono-unsaturated fatty acid; PCA, principal component analysis; PPAR: peroxisome proliferator-activated receptors; SFA, saturated fatty acid

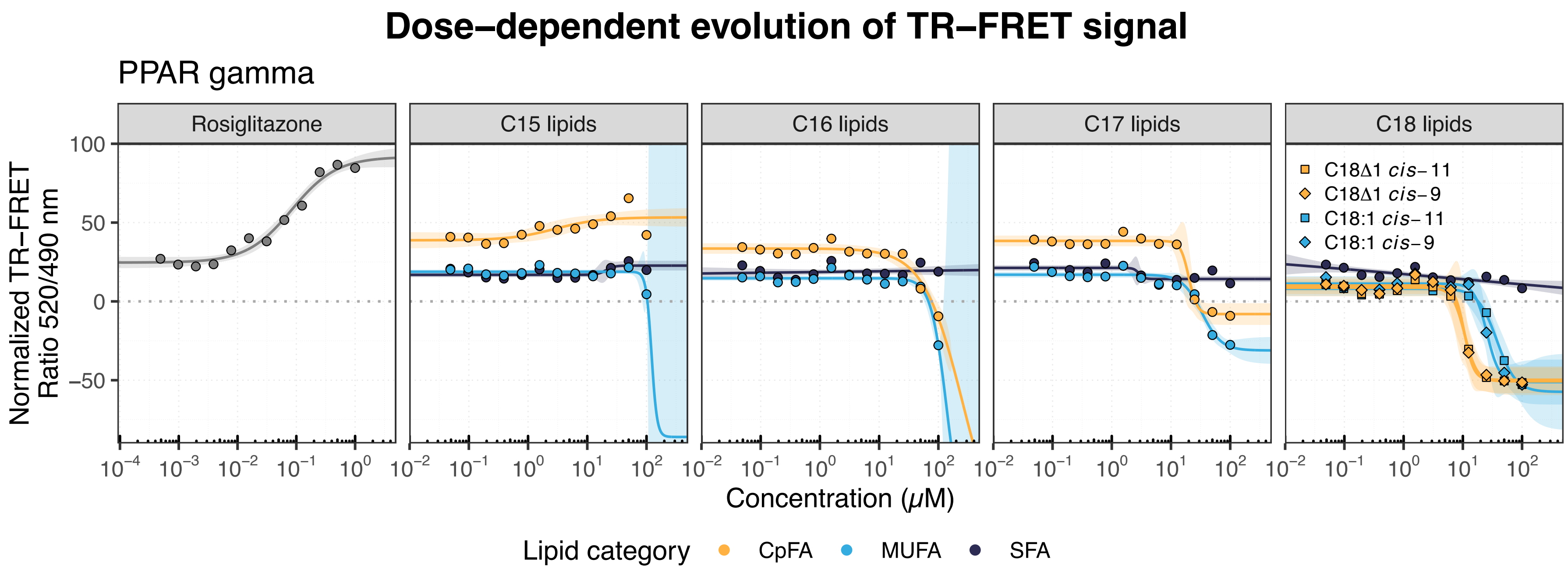

Supplementary Figure 6. PPARγ binding results using TR-FRET binding assays.

CpFA, cyclopropane fatty acids; EC50: half maximal effective concentration; Max: maximum fitted intensity; MUFA, mono-unsaturated fatty acid; PCA, principal component analysis; PPAR: peroxisome proliferator-activated receptors; SFA, saturated fatty acid

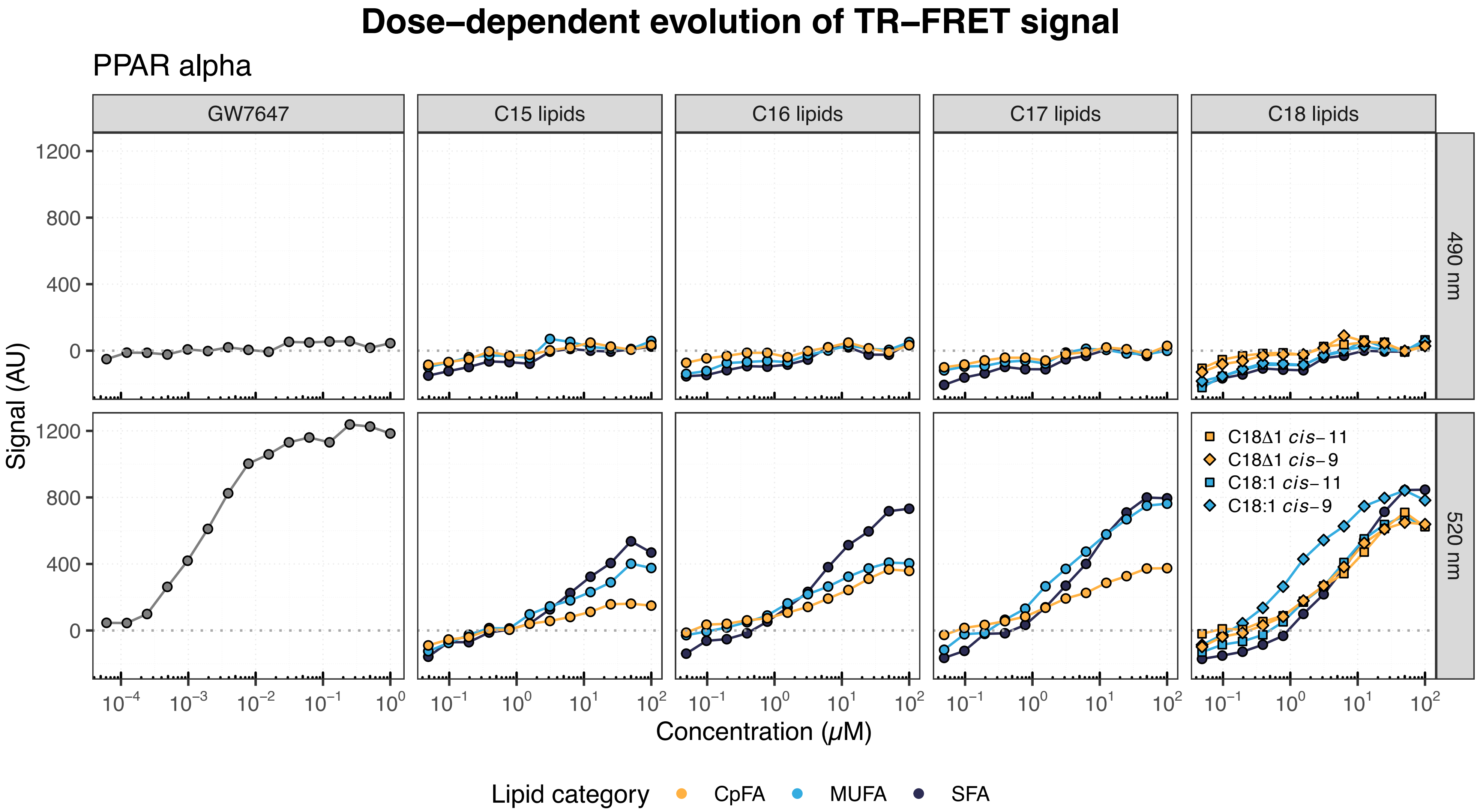

Supplementary Figure 7. 490 nm and 520 nm responses for the PPARα TR-FRET binding assay.

CpFA, cyclopropane fatty acids; MUFA, mono-unsaturated fatty acid; PPAR: peroxisome proliferator-activated receptors; SFA, saturated fatty acid

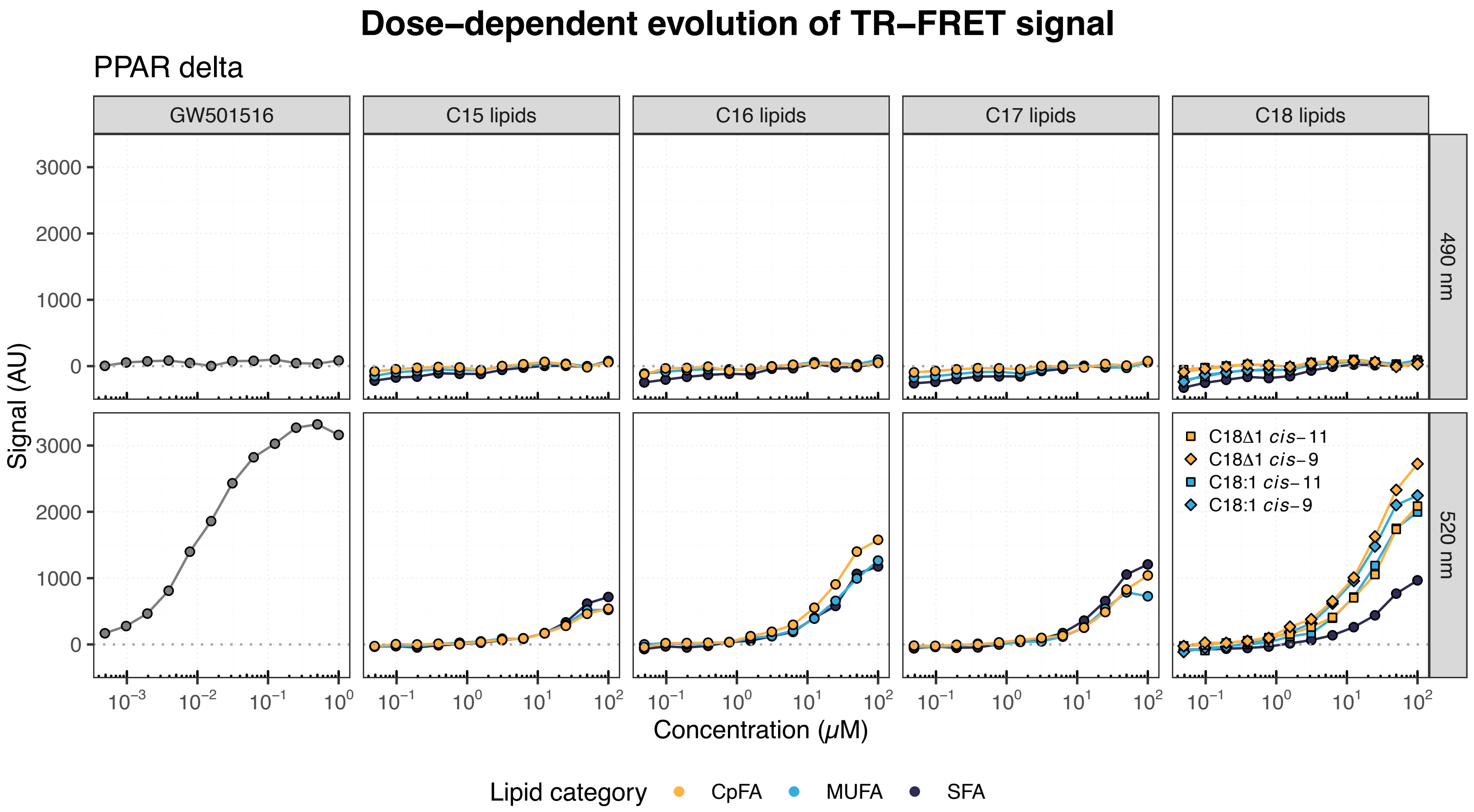

Supplementary Figure 8. 490 nm and 520 nm responses for the PPARδ TR-FRET binding assay.

CpFA, cyclopropane fatty acids; MUFA, mono-unsaturated fatty acid; PPAR: peroxisome proliferator-activated receptors; SFA, saturated fatty acid

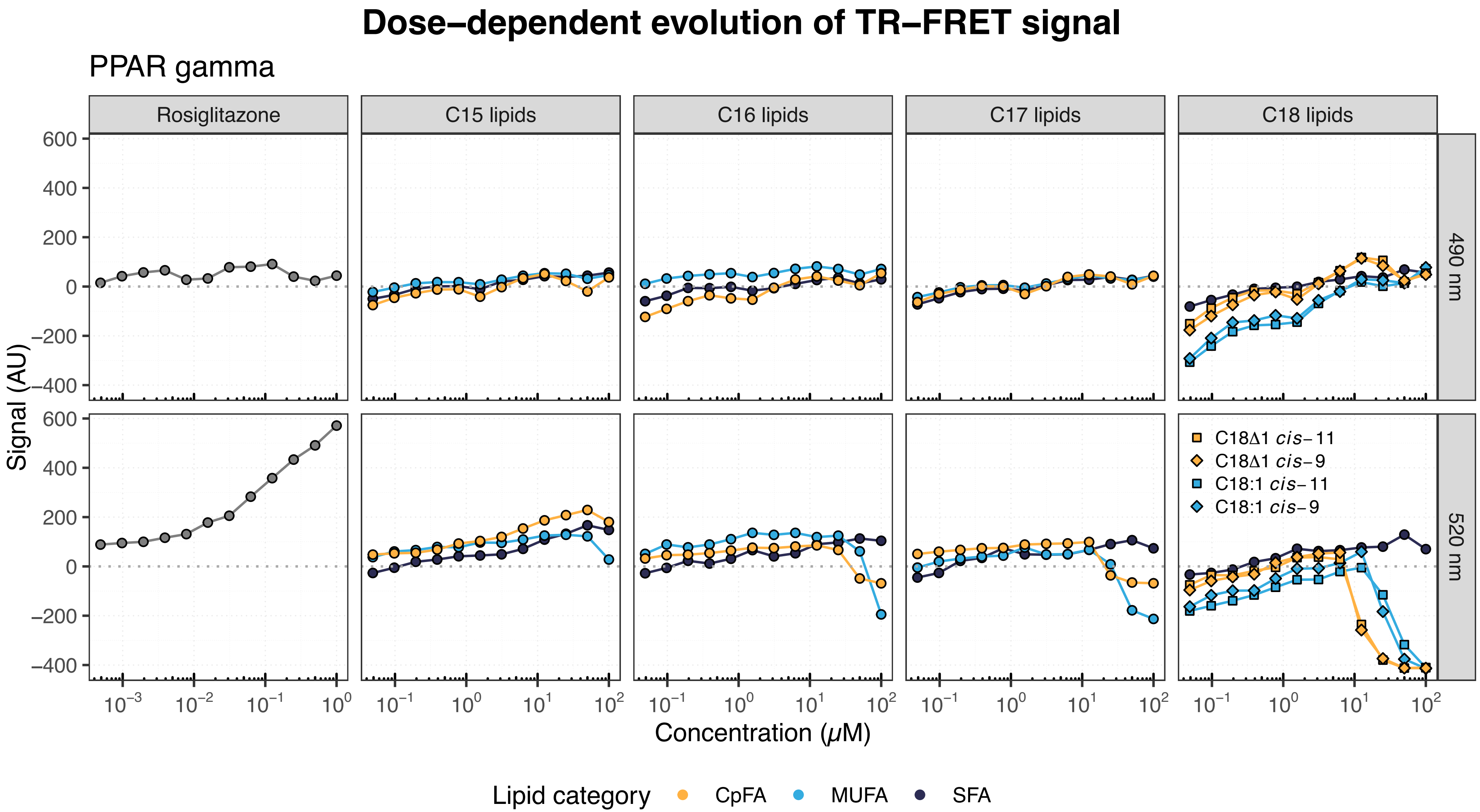

Supplementary Figure 9. 490 nm and 520 nm responses for the PPARγ TR-FRET binding assay.

CpFA, cyclopropane fatty acids; MUFA, mono-unsaturated fatty acid; PPAR: peroxisome proliferator-activated receptors; SFA, saturated fatty acid

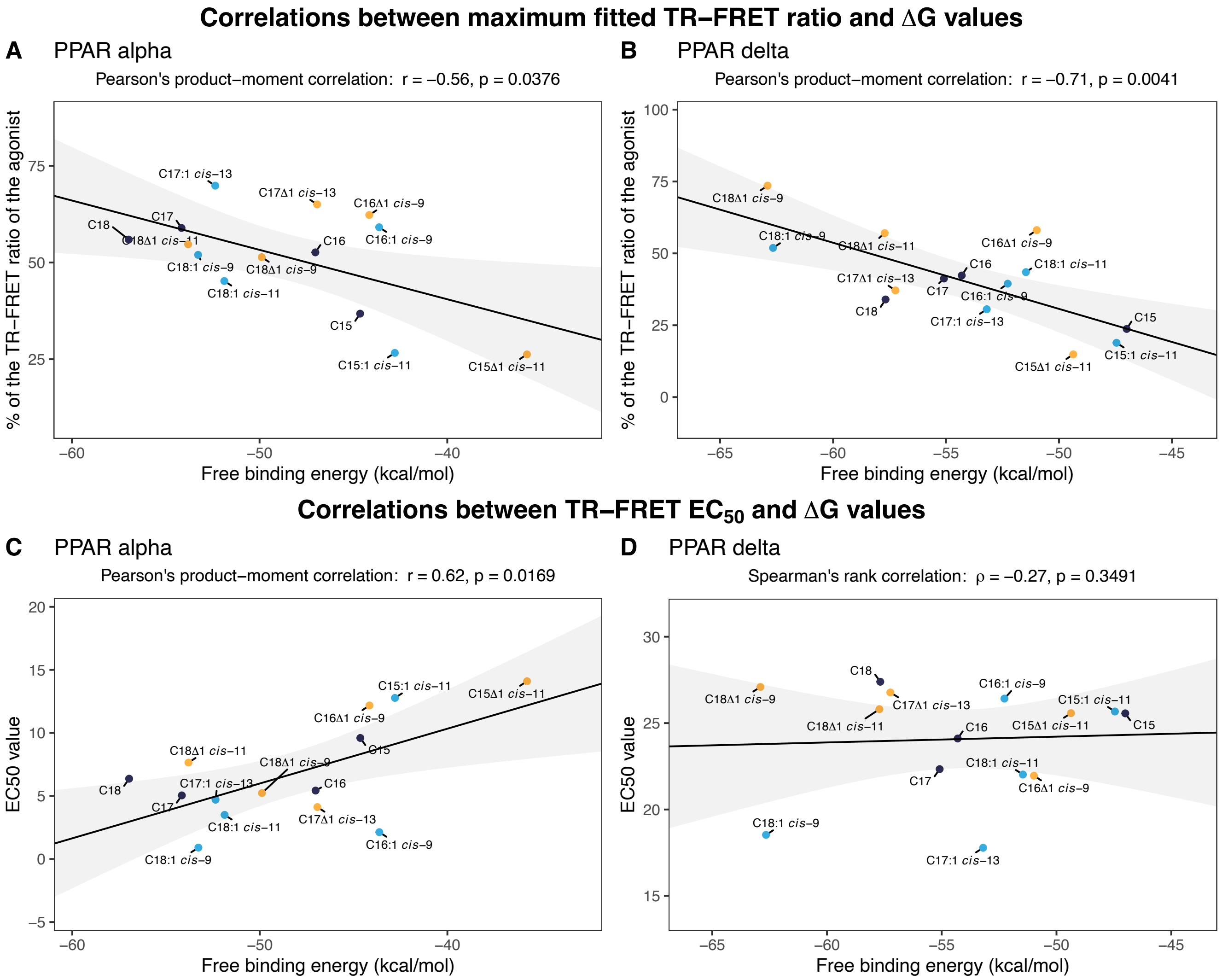

Supplementary Figure 10. In vitro binding results mostly corroborate predicted binding affinities obtained in silico.

(A-B) Pearson's correlations between in vitro Emax values and in silico binding energies for PPARα and PPARδ, respectively. (C-D) Pearson's and Spearman's correlations between in vitro EC50 values and in silico binding energies for PPARα and PPARδ, respectively.

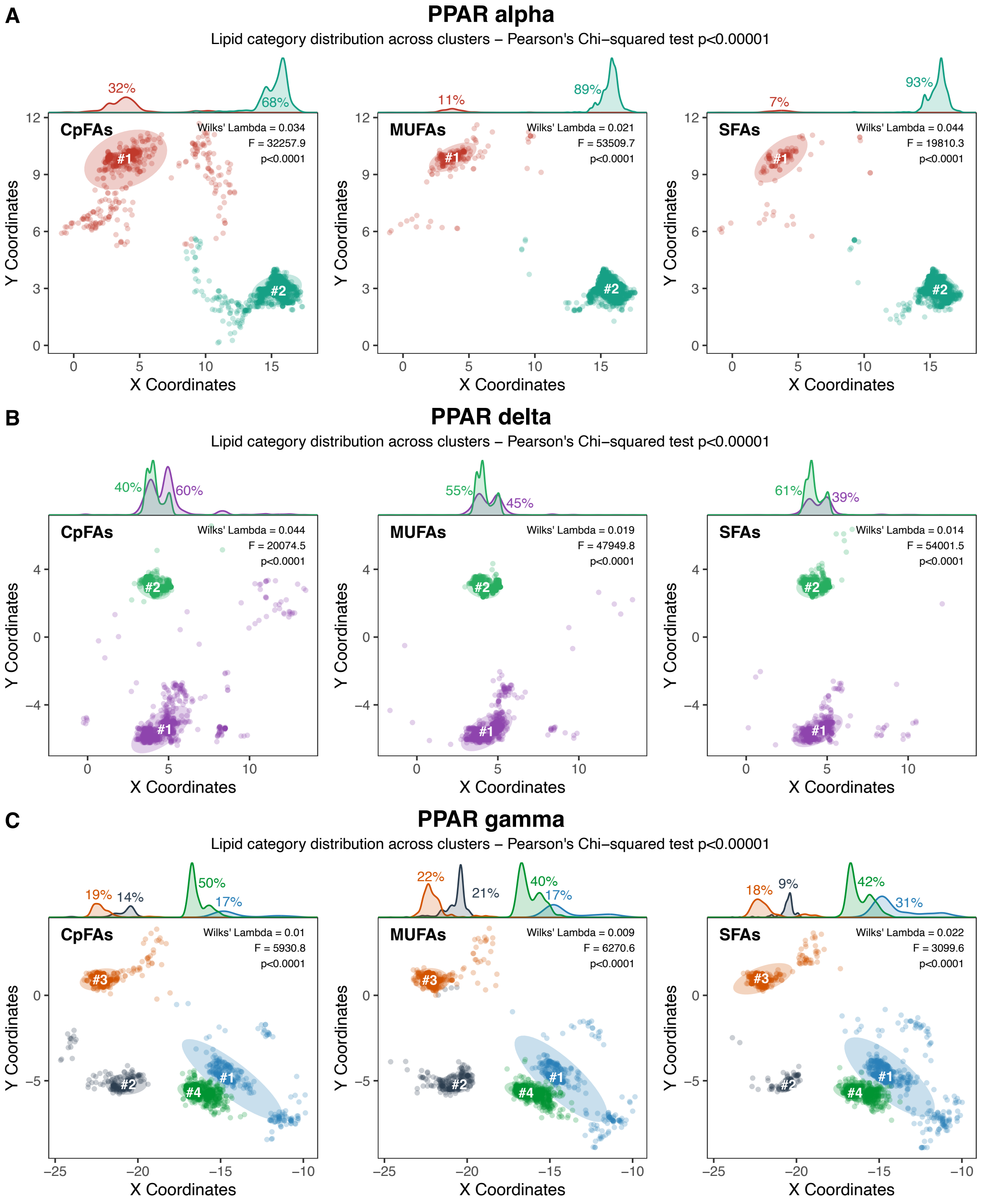

Supplementary Figure 11. Distribution of lipid categories within conformational clusters per PPAR isoform

(A–C) Two-dimensional coordinates of head-group positions for all conformers generated from the 14 lipids tested in vitro (see Supplementary Table 3), shown separately for CpFAs (left), MUFAs (middle), and SFAs (right) within the ligand-binding domains of PPARα (A), PPARδ (B), and PPARγ (C). Conformers are grouped by spatial proximity using k-means clustering. Density plots above each panel depict the relative distribution of conformers from each cluster within a given lipid category. Lipid category enrichment across clusters was evaluated using Pearson's chi-squared test, and the degree of separation between clusters was assessed via MANOVA. This visualization highlights receptor-specific clustering patterns associated with each lipid class.

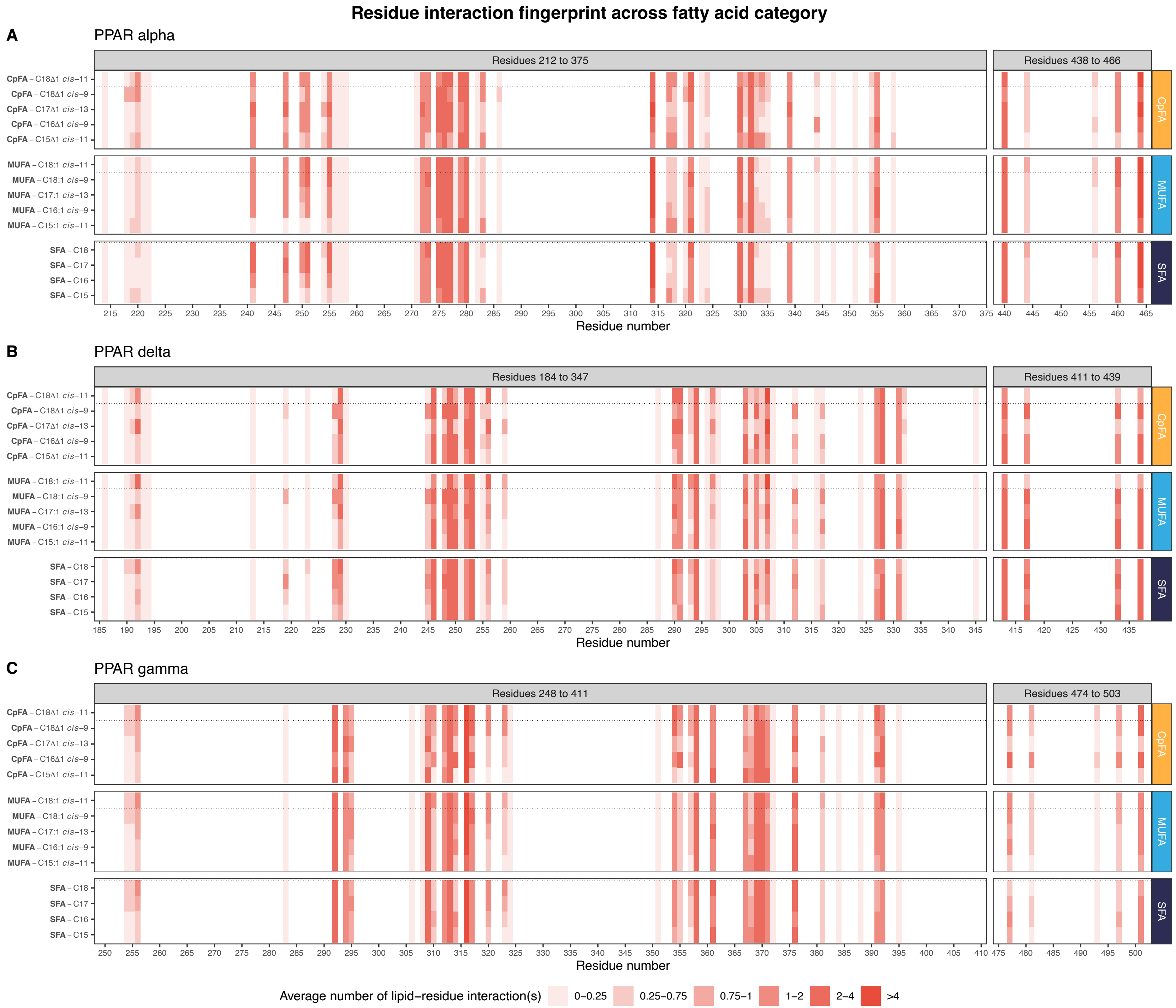

Supplementary Figure 12. Residue interaction fingerprint across fatty acid category.

(A-C) Heatmap showing the average number of lipid-residue interaction(s) for all 14 lipids tested in vitro (see Supplementary Table 3), categorized per lipid class (CpFA, MUFA and SFA), for each ligand-binding domain of PPARα (A), PPARδ (B), and PPARγ (C). The total interaction count per residue represents the sum of nine interaction types: Contact, Backbone, Sidechain, Polar, Hydrophobic, Acceptor, Donor, Aromatic, and Charged. Interaction values were binned into intervals for visual clarity. White indicates the absence of detectable interactions.

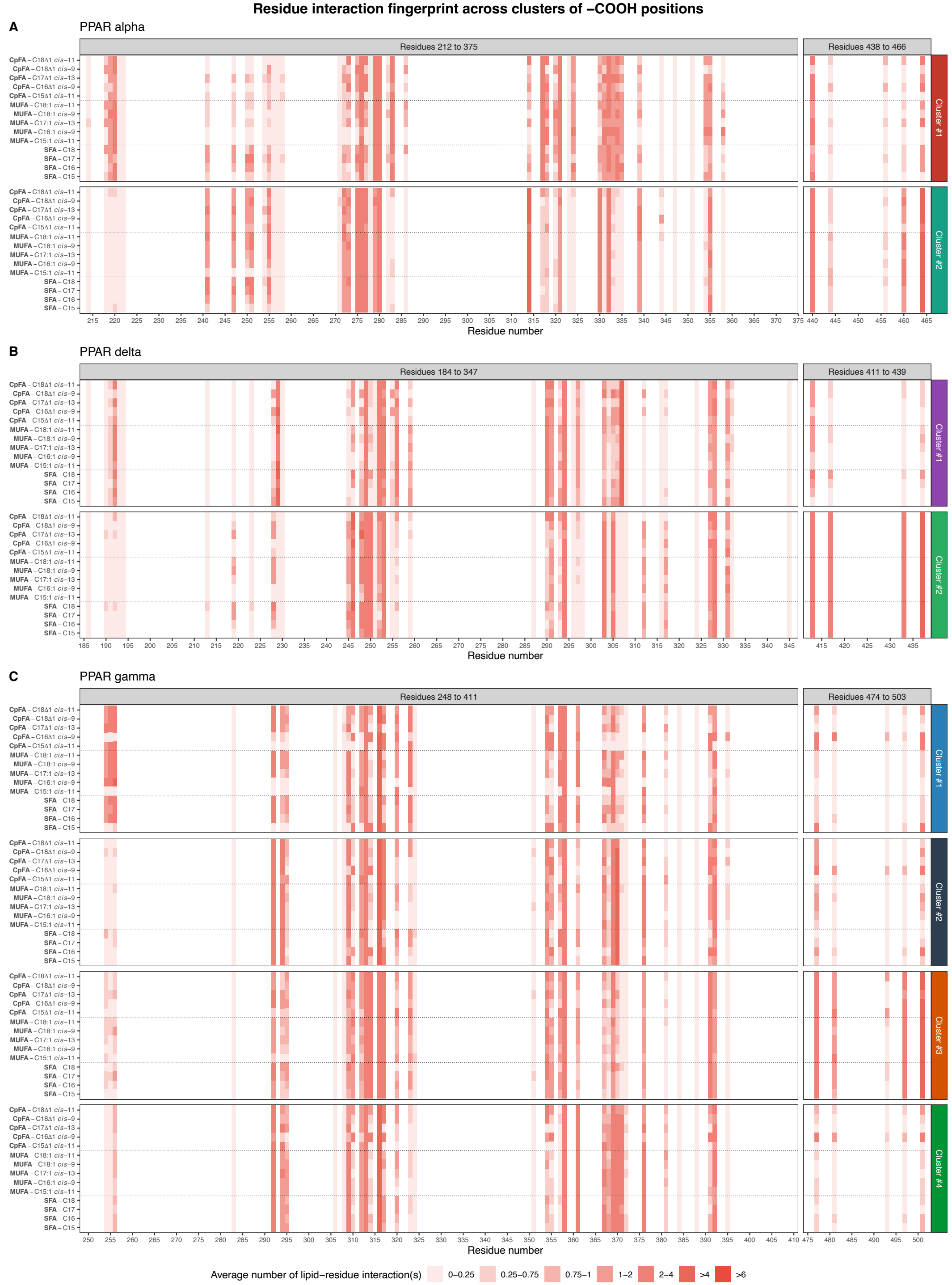

Supplementary Figure 13. Residue interaction fingerprint across clusters of lipid positions within the receptors' LBDs.

(A-C) Heatmap showing the average number of lipid-residue interaction(s) for all 14 lipids tested in vitro (see Supplementary Table 3), categorized per clusters of lipid positions, for each ligand-binding domain of PPARα (A), PPARδ (B), and PPARγ (C). The total interaction count per residue represents the sum of nine interaction types: Contact, Backbone, Sidechain, Polar, Hydrophobic, Acceptor, Donor, Aromatic, and Charged. Interaction values were binned into intervals for visual clarity. White indicates the absence of detectable interactions.

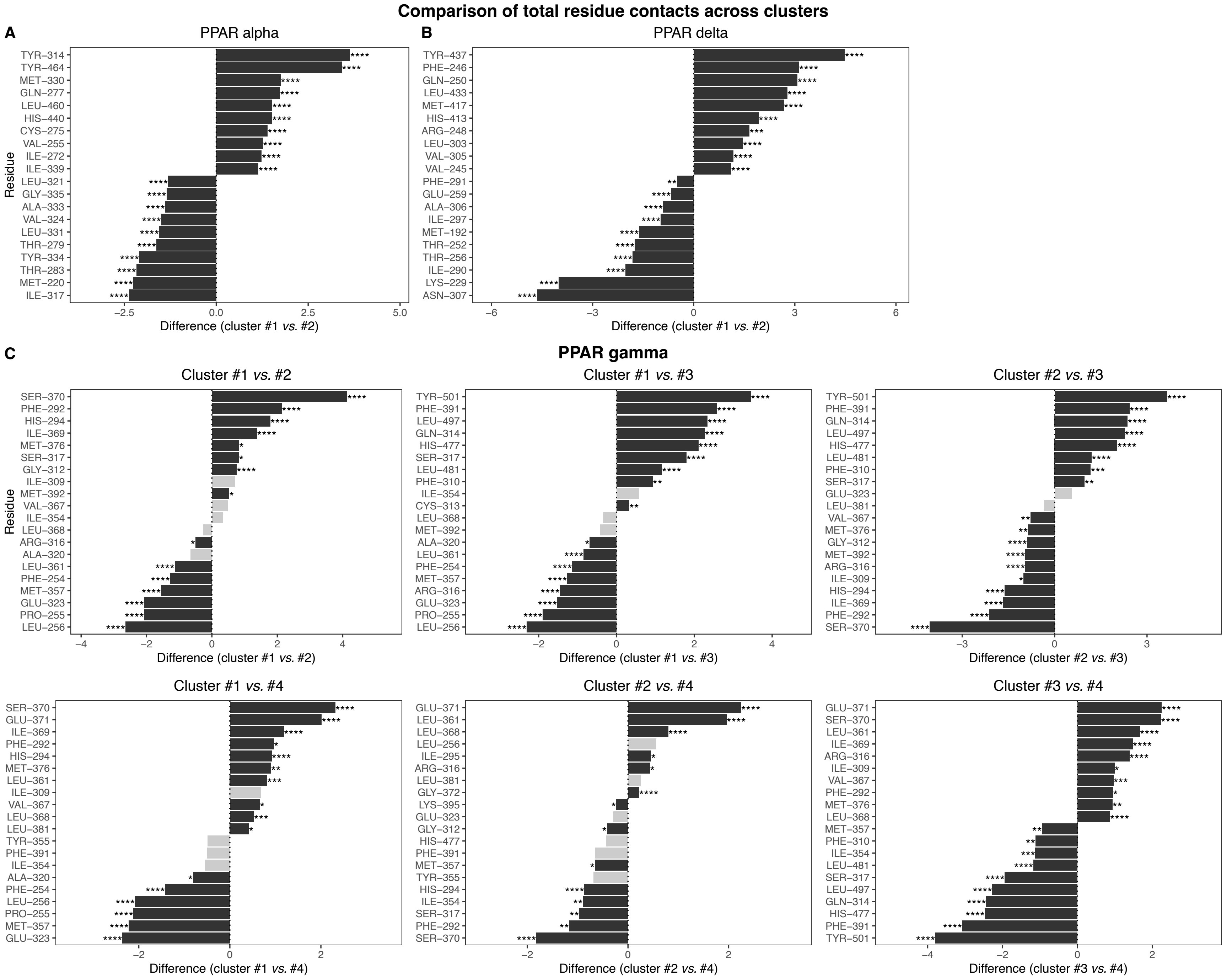

Supplementary Figure 14. Differential residue interaction patterns across structural clusters for each PPAR isoform

Bar plots displaying the signed differences in average number of lipid-residue interaction scores between selected cluster pairs for PPARα, PPARδ, and PPARγ, based on all 14 lipids tested in vitro. Each bar represents a residue and corresponds to the difference in mean interaction score between two clusters (positive = stronger in the second cluster; negative = stronger in the first). Interaction scores represent the sum of nine interaction types: Contact, Backbone, Sidechain, Polar, Hydrophobic, Acceptor, Donor, Aromatic, and Charged. Panels (A-B) show the comparison between cluster #1 and #2 for PPARα and PPARδ, respectively. Panel C display all six pairwise comparisons between clusters #1-#4 for PPARγ. Bars are colored by statistical significance after FDR correction (dark grey: $q < 0.05$; light grey: not significant). Only the top 20 residues with the largest absolute differences are shown for each comparison.

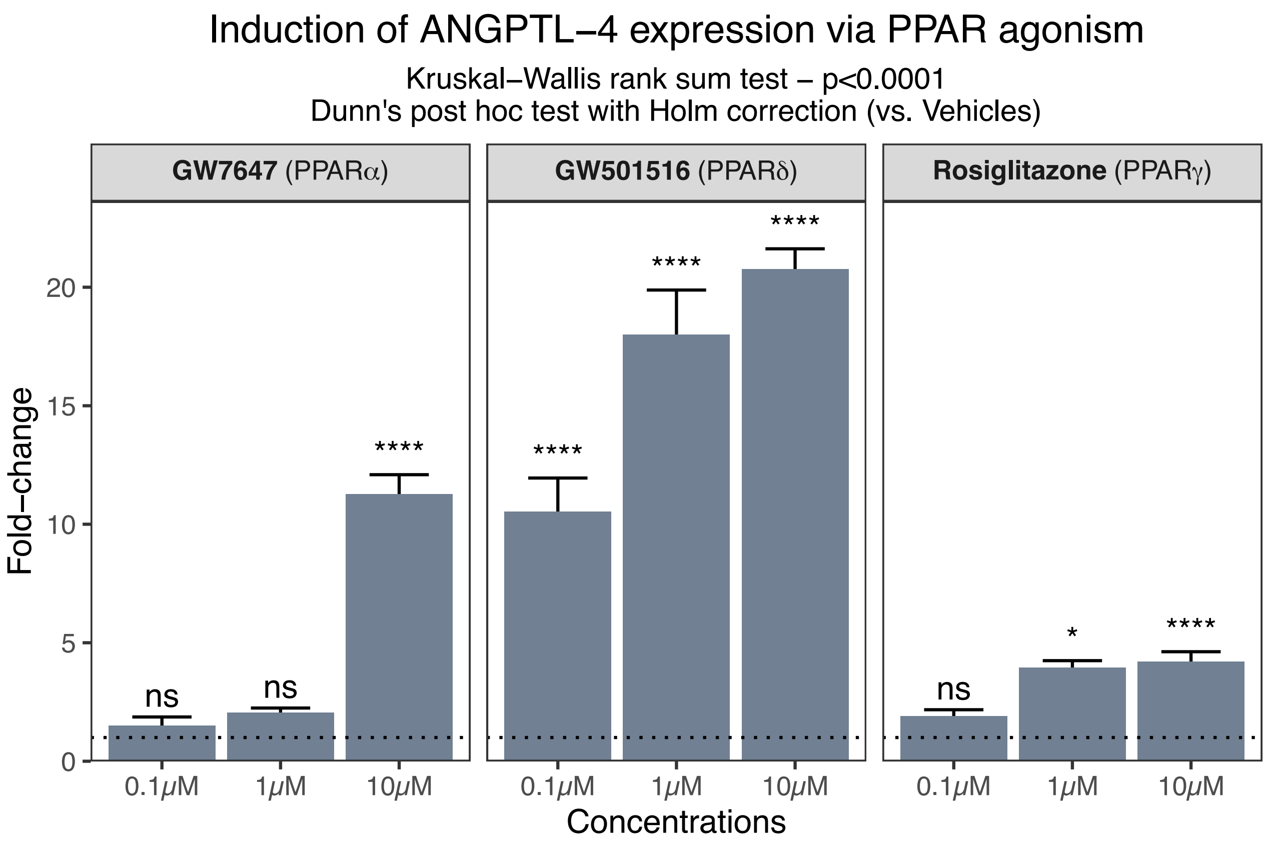

Supplementary Figure 15. Dose-dependent induction of Angptl-4 expression by PPAR agonists in 3T3-L1 preadipocyte cells

Bar-plots showing Angptl-4 mRNA fold-change following treatment with increasing concentrations (0.1, 1, and 10 μM) of selective agonists for PPARα (GW7647), PPARδ (GW501516), and PPARγ (Rosiglitazone). Gene expression was measured by RT-qPCR and normalized to vehicle-treated controls. Statistical significance was assessed using Kruskal–Wallis rank sum tests, followed by Dunn's post hoc tests with Holm correction versus vehicle. Asterisks indicate significance levels: $p < 0.05$ (*), < 0.0001 (***); ns = not significant. Error bars represent standard error of the mean (SEM). Angptl-4, Angiopoietin-like protein 4; PPAR, peroxisome proliferator-activated receptors.

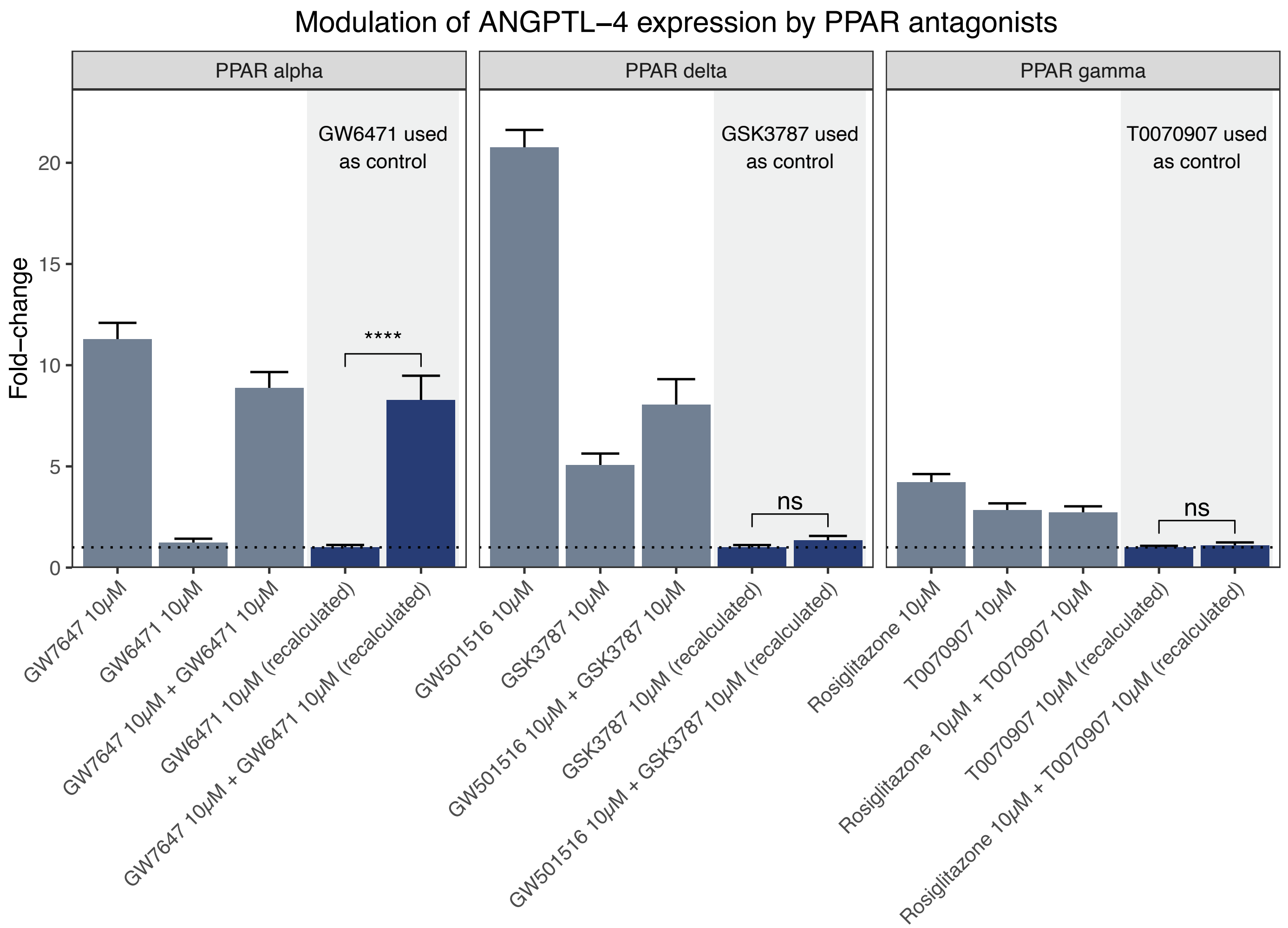

Supplementary Figure 16. Repression of Angptl-4 expression by PPAR antagonists in 3T3-L1 preadipocyte cells

Bar-plots showing Angptl-4 mRNA fold-change in response to PPAR agonists alone or in combination with their respective antagonists for PPARα (GW7647 with GW6471), PPARδ (GW501516 with GSK3787), and PPARγ (Rosiglitazone with T0070907). Both agonists and antagonists were used at 10 μM. Conditions with a light grey background are normalized to their respective vehicle controls, while those with navy blue backgrounds represent recalculated values normalized against the antagonist-only condition. Error bars represent standard error of the mean (SEM). Angptl-4, Angiopoietin-like protein 4; PPAR, peroxisome proliferator-activated receptors.
